# Supplementary material for: Objective differences and pathway differentiation between twin subtypes within the same IDDSI level: A texture–nutrition analysis based on commonly consumed foods among Chinese elderly
Source: PLoS One. 2026 Apr 8;21(4):e0344507. doi: 10.1371/journal.pone.0344507 (PMC13061176; doi:10.1371/journal.pone.0344507)
Supplement: Attachment — Submitted filename: PONE-D-25-62691_reviewer.pdf [file pone.0344507.s002.pdf]

PLOS ONE

Objective Differences and Pathway Differentiation between Twin Subtypes within the Same IDDSI Level: A Texture–Nutrition Analysis Based on Commonly Consumed Foods among Chinese Elderly

--Manuscript Draft--

**Manuscript Number:**	PONE-D-25-62691
**Article Type:**	Research Article
**Full Title:**	Objective Differences and Pathway Differentiation between Twin Subtypes within the Same IDDSI Level: A Texture–Nutrition Analysis Based on Commonly Consumed Foods among Chinese Elderly
**Short Title:**	Objective Differences and Pathway Differentiation between Twin Subtypes within the Same IDDSI Level
**Corresponding Author:**	Muxi Chen<br>Sichuan University West China Hospital: West China Hospital of Sichuan University<br>Chengdu, 四川 CHINA
**Keywords:**	Dysphagia; IDDSI; Texture analysis; Pathway differentiation; Elderly nutrition; Twin subtype
**Abstract:**	Objective<br><br>This study adopts a “pathway-priority” perspective to systematically quantify the texture–moisture–nutrition continuum of elderly-preferred ingredients commonly consumed in China under the International Dysphagia Diet Standardisation Initiative (IDDSI) framework. It focuses on intra-level differentiation between “twin-type/pathway pairs” (MO3 vs. LQ3, EX4 vs. PU4, EC7 vs. RG7), aiming to reveal objective distinctions in texture and nutritional orientation across pathways and to provide quantitative evidence for dietary level classification and prescription substitution in dysphagia management.<br><br>Methods<br><br>Twenty-six representative ingredients were collected from seven major Chinese dietary regions through Python-based web crawling. According to IDDSI classification, samples were grouped into the liquid pathway (Levels 0–2, MO3, EX4) and food pathway (LQ3, PU4, Levels 5–7 including EC7/RG7). A TA.New Plus texture analyzer was used to measure hardness, adhesiveness, and cohesiveness. Nutritional components per 100 g—including energy, protein, fat, carbohydrate, and dietary fiber—were calculated using Huaxi Hospital Nutrition software. Statistical analyses included Mann–Whitney U tests with Cliff’s δ for twin-type comparisons, Spearman correlation and Theil–Sen slope analysis for monotonic trends within pathways, and Kruskal–Wallis followed by Dunn–Bonferroni tests for intra-level differences among food categories. False discovery rate (FDR–BH) correction was applied, with significance set at $q \le 0.05$.<br><br>Results<br><br>(1) In twin-type comparisons, MO3, EX4, and EC7 exhibited significantly lower hardness and energy density but higher moisture content ($q \le 0.05$), whereas LQ3, PU4, and RG7 contained higher energy and protein levels, reflecting hydration-safety orientation in liquid pathways versus structural-nutritional orientation in food pathways.(2) Within-pathway analyses showed that the liquid pathway (Levels 0→4) followed a “thickening–hardening–dehydrating” monotonic trend, with increasing hardness and energy but decreasing adhesiveness and moisture. The food pathway (Levels 3→7) displayed a “structuring–hardening–dehydrating–energizing” trajectory, indicating enhanced granularity and nutritional concentration.(3) Significant texture differences among food categories within the same level were observed (ε^2 = 0.37–0.67, $q \le 0.05$), suggesting that “same level ≠ interchangeable.”(4) The established “safety–nutrition balance window” identified key control zones: moisture 88.9–96.8 g/100 g and hardness 100–160 N/m^2 for MO3/EX4, and energy 70–324

Powered by Editorial Manager® and ProduXion Manager® from Aries Systems Corporation

	kcal/100 g for PU4/RG7, providing practical references for clinical and process optimization.<br><br>Conclusion<br><br>Twin subtypes within the same IDDSI level exhibit systematic differences in texture and nutrition, confirming the hypothesis that “same level ≠ interchangeable.” The liquid pathway emphasizes safety and hydration, while the food pathway prioritizes structure and nutrient density. By constructing an intra-pathway “texture–nutrition continuum” and safety–nutrition balance window, this study offers quantitative evidence for pathway selection, diet prescription, and quality control in dysphagia management. Future work should integrate VFSS/FEES imaging and clinical outcomes to map laboratory metrics to swallowing safety, facilitating translation from experimental assessment to clinical application.
**Order of Authors:**	Muxi Chen
	Yi Cheng
	Juan Duan
	Lei Shi
	Liu Yuan
**Additional Information:**	
**Question**	**Response**
**Financial Disclosure**<br><br>Enter a financial disclosure statement that describes the sources of funding for the work included in this submission. Review the submission guidelines for detailed requirements. View published research articles from *PLOS ONE* for specific examples.<br><br>This statement is required for submission and **will appear in the published article** if the submission is accepted. Please make sure it is accurate.<br><br>**Funded studies**<br>Enter a statement with the following details:<br>• Initials of the authors who received each award<br>• Grant numbers awarded to each author<br>• The full name of each funder<br>• URL of each funder website<br>• Did the sponsors or funders play any role in the study design, data collection and analysis, decision to publish, or preparation of the manuscript?<br><br>Did you receive funding for this work?	Yes

Powered by Editorial Manager® and ProduXion Manager® from Aries Systems Corporation

<table>
<tr>
<td>Please add funding details.
as follow-up to "Financial Disclosure

Enter a financial disclosure statement that describes the sources of funding for the work included in this submission. Review the submission guidelines for detailed requirements. View published research articles from PLOS ONE for specific examples.

This statement is required for submission and will appear in the published article if the submission is accepted. Please make sure it is accurate.

Funded studies
Enter a statement with the following details:
• Initials of the authors who received each award
• Grant numbers awarded to each author
• The full name of each funder
• URL of each funder website
• Did the sponsors or funders play any role in the study design, data collection and analysis, decision to publish, or preparation of the manuscript?

Did you receive funding for this work?"</td>
<td>Funding：Y.L. received funding from the project "Key Technologies and New Product Development for Intelligent and Safe Production of Foods for Special Medical Purposes" (Grant No. JH2019045).L.S. received funding from the project "Development and Industrialization Demonstration of Nutrition and Health Foods for the Elderly" (Grant No. 2023YFF1104405), funded by the Ministry of Science and Technology of China.</td>
</tr>
<tr>
<td>Please select the country of your main research funder (please select carefully as in some cases this is used in fee calculation).
as follow-up to "Financial Disclosure

Enter a financial disclosure statement that describes the sources of funding for the work included in this submission. Review the submission guidelines for detailed requirements. View published research articles from PLOS ONE for specific examples.

This statement is required for submission and will appear in the published article if the submission is accepted. Please make sure it is accurate.</td>
<td>CHINA - CN</td>
</tr>
</table>

**Funded studies**<br>Enter a statement with the following details:<br>• Initials of the authors who received each award<br>• Grant numbers awarded to each author<br>• The full name of each funder<br>• URL of each funder website<br>• Did the sponsors or funders play any role in the study design, data collection and analysis, decision to publish, or preparation of the manuscript?<br><br>Did you receive funding for this work?"	
**Competing Interests**<br><br>Use the instructions below to enter a competing interest statement for this submission. On behalf of all authors, disclose any competing interests that could be perceived to bias this work—acknowledging all financial support and any other relevant financial or non-financial competing interests.<br><br>This statement is required for submission and **will appear in the published article** if the submission is accepted. Please make sure it is accurate and that any funding sources listed in your Funding Information later in the submission form are also declared in your Financial Disclosure statement.<br><br>View published research articles from *PLOS ONE* for specific examples.	The authors have declared that no competing interests exist.

Powered by Editorial Manager® and ProduXion Manager® from Aries Systems Corporation

**NO authors have competing interests**<br><br>Enter: *The authors have declared that no competing interests exist.*<br><br>**Authors with competing interests**<br><br>Enter competing interest details beginning with this statement:<br><br>*I have read the journal's policy and the authors of this manuscript have the following competing interests: [insert competing interests here]*<br><br>* typeset	
**Ethics Statement**<br><br>Enter an ethics statement for this submission. This statement is required if the study involved:<br><br>• Human participants<br>• Human specimens or tissue<br>• Vertebrate animals or cephalopods<br>• Vertebrate embryos or tissues<br>• Field research<br><br>Write "N/A" if the submission does not require an ethics statement.<br><br>General guidance is provided below. Consult the submission guidelines for detailed instructions. **Make sure that all information entered here is included in the Methods section of the manuscript.**	This study did not involve any human participants or animal experiments. All food materials were commercially sourced and handled in accordance with the Laboratory Food Safety Code of Practice of West China Hospital, Sichuan University. Therefore, ethical approval and informed consent were not required.

**Format for specific study types**<br><br>**Human Subject Research (involving human participants and/or tissue)**<br>• Give the name of the institutional review board or ethics committee that approved the study<br>• Include the approval number and/or a statement indicating approval of this research<br>• Indicate the form of consent obtained (written/oral) or the reason that consent was not obtained (e.g. the data were analyzed anonymously)<br><br>**Animal Research (involving vertebrate animals, embryos or tissues)**<br>• Provide the name of the Institutional Animal Care and Use Committee (IACUC) or other relevant ethics board that reviewed the study protocol, and indicate whether they approved this research or granted a formal waiver of ethical approval<br>• Include an approval number if one was obtained<br>• If the study involved *non-human primates*, add *additional details* about animal welfare and steps taken to ameliorate suffering<br>• If anesthesia, euthanasia, or any kind of animal sacrifice is part of the study, include briefly which substances and/or methods were applied<br><br>**Field Research**<br><br>Include the following details if this study involves the collection of plant, animal, or other materials from a natural setting:<br>• Field permit number<br>• Name of the institution or relevant body that granted permission	
**Data Availability**<br><br>Authors are required to make all data underlying the findings described fully available, without restriction, and from the time of publication. PLOS allows rare exceptions to address legal and ethical concerns. See the PLOS Data Policy and FAQ for detailed information.	Yes - all data are fully available without restriction

A Data Availability Statement describing where the data can be found is required at submission. Your answers to this question constitute the Data Availability Statement and **will be published in the article**, if accepted.<br><br>**Important:** Stating 'data available on request from the author' is not sufficient. If your data are only available upon request, select 'No' for the first question and explain your exceptional situation in the text box.<br><br>Do the authors confirm that all data underlying the findings described in their manuscript are fully available without restriction?	
**Describe where the data may be found in full sentences. If you are copying our sample text, replace any instances of XXX with the appropriate details.**<br><br>• If the data are **held or will be held in a public repository**, include URLs, accession numbers or DOIs. If this information will only be available after acceptance, indicate this by ticking the box below. For example: *All XXX files are available from the XXX database (accession number(s) XXX, XXX.).*<br>• If the data are all contained **within the manuscript and/or Supporting Information files**, enter the following: *All relevant data are within the manuscript and its Supporting Information files.*<br>• If neither of these applies but you are able to provide **details of access elsewhere**, with or without limitations, please do so. For example:<br><br>*Data cannot be shared publicly because of [XXX]. Data are available from the XXX Institutional Data Access / Ethics Committee (contact via XXX) for researchers who meet the criteria for access to confidential data.*<br><br>*The data underlying the results presented in the study are available from (include the name of the third party*	All relevant data are within the manuscript and its Supporting Information files.

and contact information or URL).<br>• This text is appropriate if the data are owned by a third party and authors do not have permission to share the data.<br><br>* typeset	
Additional data availability information:	Tick here if the URLs/accession numbers/DOIs will be available only after acceptance of the manuscript for publication so that we can ensure their inclusion before publication.

Manuscript
Click here to access/download;Manuscript;11-24Objective Differences and Pathway Differentiation.docx

Objective Differences and Pathway Differentiation between Twin Subtypes within the Same IDDSI Level: A Texture–Nutrition Analysis Based on Commonly Consumed Foods among Chinese Elderly

Muxi Chen[1,2], Yi Cheng[1],Juan Duan[1], Lei Shi[1], Liu Yuan[1*]

1.Department of Clinical Nutrition, West China Hospital, Sichuan University, Chengdu, 610041, China

2. Clinical Medicine College, College of Traditional Chinese Medicine, Mianyang 621000, China

*Correspondence: liu63415@163.com

Funding : Y.L. received funding from the project "Key Technologies and New Product Development for Intelligent and Safe Production of Foods for Special Medical Purposes" (Grant No. JH2019045).L.S. received funding from the project "Development and Industrialization Demonstration of Nutrition and Health Foods for the Elderly" (Grant No. 2023YFF1104405), funded by the Ministry of Science and Technology of China.

Abstract

Objective:

This study adopts a “pathway-priority” perspective to systematically quantify the texture–moisture–nutrition continuum of elderly-preferred ingredients commonly consumed in China under the *International Dysphagia Diet Standardisation Initiative* (IDDSI) framework. It focuses on intra-level differentiation between “twin-type/pathway pairs” (MO3 vs. LQ3, EX4 vs. PU4, EC7 vs. RG7), aiming to reveal objective distinctions in texture and nutritional orientation across pathways and to provide quantitative evidence for dietary level classification and prescription substitution in dysphagia management.

Methods:

Twenty-six representative ingredients were collected from seven major Chinese dietary regions through Python-based web crawling. According to IDDSI classification, samples were grouped into the *liquid pathway* (Levels 0–2, MO3, EX4) and *food pathway* (LQ3, PU4, Levels 5–7 including EC7/RG7). A TA.New Plus texture analyzer was used to measure hardness, adhesiveness, and cohesiveness. Nutritional components per 100 g—including energy, protein, fat, carbohydrate, and dietary fiber—were calculated using *Huaxi Hospital Nutrition* software. Statistical analyses included Mann–Whitney U tests with Cliff’s δ for twin-type comparisons, Spearman correlation and Theil–Sen slope analysis for monotonic trends within pathways, and Kruskal–Wallis followed by Dunn–Bonferroni tests for intra-level differences among food categories. False discovery rate (FDR–BH) correction was applied, with significance set at $q \le 0.05$.

Results:

(1) In twin-type comparisons, MO3, EX4, and EC7 exhibited significantly lower

hardness and energy density but higher moisture content ($q \leq 0.05$), whereas LQ3, PU4, and RG7 contained higher energy and protein levels, reflecting hydration-safety orientation in liquid pathways versus structural-nutritional orientation in food pathways.
(2) Within-pathway analyses showed that the liquid pathway (Levels 0→4) followed a “thickening–hardening–dehydrating” monotonic trend, with increasing hardness and energy but decreasing adhesiveness and moisture. The food pathway (Levels 3→7) displayed a “structuring–hardening–dehydrating–energizing” trajectory, indicating enhanced granularity and nutritional concentration.
(3) Significant texture differences among food categories within the same level were observed ($\varepsilon^2 = 0.37$–0.67, $q \leq 0.05$), suggesting that “same level ≠ interchangeable.”
(4) The established “safety–nutrition balance window” identified key control zones: moisture 88.9–96.8 g/100 g and hardness 100–160 N/m^2 for MO3/EX4, and energy 70–324 kcal/100 g for PU4/RG7, providing practical references for clinical and process optimization.

Conclusion:
Twin subtypes within the same IDDSI level exhibit systematic differences in texture and nutrition, confirming the hypothesis that “same level ≠ interchangeable.” The liquid pathway emphasizes safety and hydration, while the food pathway prioritizes structure and nutrient density. By constructing an intra-pathway “texture–nutrition continuum” and safety–nutrition balance window, this study offers quantitative evidence for pathway selection, diet prescription, and quality control in dysphagia management. Future work should integrate VFSS/FEES imaging and clinical outcomes to map laboratory metrics to swallowing safety, facilitating translation from experimental assessment to clinical application.

Keywords:
Dysphagia; IDDSI; Texture analysis; Pathway differentiation; Elderly nutrition; Twin subtype

1. Introduction

China has rapidly entered an aging society, accompanied by growing health demands among older adults. Swallowing function gradually declines during aging and under chronic conditions such as stroke, Parkinson's disease, Alzheimer's disease, and post–head and neck cancer therapy, leading to varying degrees of dysphagia. The prevalence of dysphagia increases markedly with age and is particularly high among specific patient populations. Studies have reported a prevalence of 66.0% among elderly Chinese, including 21.0% at ages 60–69, 28.0% at ages 70–79, and 41.0% at ≥80 years[1]. Dysphagia occurs in 50–60% of patients following head and neck cancer treatment and in 37–78% of post-stroke patients[2-3]. Dysphagia alters dietary structure and eating patterns, impairs hydration and nutrient intake, and is closely associated with aspiration pneumonia, malnutrition, frailty, and reduced quality of life[4]. Within the continuum of "healthy aging" and integrated home–institution–medical care, developing diets that balance safety, nutrition, and compliance across functional states has become an urgent clinical and public health challenge.

To standardize terminology and practice, the *International Dysphagia Diet Standardisation Initiative* (IDDSI) established an eight-level continuum (Levels 0–7) for both drinks and foods, with standardized testing for texture and consistency[5]. The drink pathway primarily covers Levels 0–4 (0: thin; 1: slightly thick; 2: mildly thick; 3: moderately thick; 4: extremely thick), while the food pathway encompasses Levels 3–7 (3: liquidized; 4: pureed; 5: minced & moist; 6: soft & bite-sized; 7: regular/easy to chew). Natural overlaps occur at Levels 3 and 4, where the same numeric level can correspond to either a drink or a food target. At Level 7, the "regular" (RG7) type is complemented by an "easy to chew" (EC7) subtype, representing a lower bite force threshold and better oral breakdown properties[6].

Importantly, a numeric level does not imply product equivalence.First, within Levels 3 and 4, two distinct testing and swallowing-kinematic pathways coexist—here termed "twin subtypes" or "dual pathways." Level 3 includes MO3 (drink pathway, moderately thick) and LQ3 (food pathway, liquidized), while Level 4 includes EX4 (drink pathway, extremely thick) and PU4 (food pathway, pureed). Level 7 includes RG7 and EC7, both food types, the latter optimized for limited mastication.Substituting products solely by numeric level neglects key differences between syringe flow tests (drinks) and spoon/fork pressure tests (foods), resulting in mismatches in aspiration risk, hydration adequacy, and energy density [7].Second, empirical and clinical evidence indicates marked heterogeneity in hardness, adhesiveness, and cohesiveness among ingredients within the same level, owing to variations in food matrix, fiber type and content, starch gelatinization/retrogradation, protein gelation, and fat emulsification [8, 9–12]. Thus, "same level" does not guarantee interchangeability. Pathway-based intra-level classification may better explain such differences and guide precise clinical selection.

In the Chinese context, elderly-preferred ingredients differ substantially in matrix composition, colloidal behavior, fiber structure, and water-holding capacity. Consequently, foods at the same IDDSI level may exhibit distinct "texture–nutrition" profiles depending on ingredient type and processing. Providing quantitative, instrument-based evidence to support IDDSI bedside testing not only addresses the uncertainty of intra-level substitution but also supports a "pathway-first, within-pathway substitution" approach for diet prescription and quality control [10, 14]. Accordingly, this study proposes a *pathway-priority* framework for IDDSI sub-classification: explicitly distinguishing the drink and food pathways within the same numeric level, focusing on twin subtypes at Levels 3, 4, and 7 (L3: MO3 vs. LQ3; L4: EX4 vs. PU4; L7: EC7 vs. RG7), and mapping the full 0–7 continuum within each pathway. Using commonly consumed and elderly-preferred Chinese ingredients, we systematically measured and compared hardness, adhesiveness (signed), cohesiveness, moisture/addition ratio, and macronutrient composition (energy, protein, fat, carbohydrate, dietary fiber), stratified by food category.
The study aimed to:
(1) quantify objective differences and pathway differentiation between twin subtypes within the same IDDSI level;
(2) construct intra-pathway "texture–nutrition" continua; and
(3) provide data-driven, exploratory evidence for practical issues such as initial feeding form (liquid-first vs. solid-first) and rehabilitation sequencing, thereby defining operable substitution boundaries for clinical and catering applications in China.

2. Materials and Methods

2.1 Ingredient Data Collection

To better understand dietary preferences among the Chinese elderly, this study used the Python programming language and its web-scraping library Scrapy to collect data from Meishi Tianxia (www.meishichina.com), a widely used Chinese culinary website. Data were extracted from elderly-recommended recipes covering seven major Chinese dietary regions. In compliance with the website's access policy and relevant legal regulations, information on major ingredients from each recipe was retrieved. The dataset was then cleaned and filtered to remove duplicates and irrelevant content, retaining only key variables (ingredient name, category, quantity, and cooking method) for subsequent analysis.

2.2 Selection of Experimental Ingredients

Using web-scraped data, 26 representative ingredients commonly consumed and recommended for older adults across China's seven dietary regions were selected.

These ingredients included:Meats: pork, beef, chicken, fish;Eggs: chicken eggs;Grains and tubers: noodles, rice, oats, potatoes, sweet potatoes, yam,

pumpkin;Vegetables: tomato, eggplant, Chinese cabbage, carrot, green cabbage, broccoli, spinach;Mixed beans: red beans, mung beans;Fruits: apple, pear, peach, banana;Other: tofu

These items are culturally representative, widely accessible, and considered elderly-friendly in the Chinese dietary context.

2.3 Food Processing and Sample Preparation

All ingredients were processed following typical cooking methods used in elderly diets to ensure standardization.General preparation involved cleaning, cutting, and steaming until fully cooked, followed by blending with varying amounts of water to produce purees with different consistencies and hardness levels.

For example, “pumpkin puree (1:2)” indicates a mass ratio of 1 part pumpkin to 2 parts water.Specific procedures were as follows:

Meat samples: Fresh pork, beef, and fish were trimmed of fascia and fat, cut into 2×2 cm cubes, minced, steamed for 30 min, and blended with different water ratios to form purees (e.g., pork puree (1:0) means no added water).

Egg samples: Hard-boiled egg yolks were mixed with varying water ratios (e.g., yolk (1:0.5) = yolk:water = 1:0.5). For steamed eggs, beaten eggs were mixed with water and steamed (e.g., steamed egg (1:2) = egg:water = 1:2).

Grains and tubers: Rice was soaked for 30 min and steamed with a 1:4 rice–water ratio; oats were cooked at 1:8; noodles were boiled for 8 min, minced, and mixed with water (1:1).

Vegetables: Cleaned and diced (2×2 cm) vegetables were steamed for 30 min and blended (e.g., carrot puree (1:0.5) = carrot:water = 1:0.5).

Mixed beans: Red and mung beans were soaked for 12 h, steamed for 30 min, and blended (e.g., red bean puree (1:2) = bean:water = 1:2).

Fruits: Peeled, cored, diced (2×2 cm) fruits were steamed or used raw and blended with water (e.g., apple puree (1:1) = apple:water = 1:1).

Other: Tofu was steamed for 15 min, then blended directly or with small amounts of water depending on the desired consistency.

All samples followed a uniform preparation protocol to ensure comparability. Three independent replicates (50 g each) were prepared per ingredient at each consistency level for IDDSI grading and texture analysis.

2.4 IDDSI Classification and Pathway Definition

(1) IDDSI Grading Criteria

Standard International Dysphagia Diet Standardisation Initiative (IDDSI) assessment tools were employed, including the IDDSI Flow Test, Fork Drip Test, and Spoon Tilt Test.

Liquids: Classified by the remaining volume (out of 10 mL) after 10 s of flow through a syringe (Levels 0–4).

Non-flowing samples: Evaluated by fork gap retention and spoon hold tests (Levels 5–7).

Level definitions were as follows:

Level 0 = Thin; Level 1 = Slightly thick (1–4 mL remaining);

Level 2 = Mildly thick (4–8 mL); Level 3 = Moderately thick / liquidized;

Level 4 = Extremely thick / pureed; Levels 5–7 = Crushable or chewable foods.

(2) Definition of Twin Subtypes (Dual Pathways)

To capture structural and rheological differences within the same numeric IDDSI levels, Levels 3, 4, and 7 were further divided into twin subtypes (dual pathways):

Level 3: MO3 (liquid pathway, moderately thick drink) vs. LQ3 (food pathway, liquidized food)

Level 4: EX4 (liquid pathway, extremely thick drink) vs. PU4 (food pathway, pureed food)

Level 7: EC7 (easy to chew) vs. RG7 (regular)

(3) Pathway Integration

To facilitate comparative and trend analyses, IDDSI levels were reorganized into two integrated pathways:

Liquid pathway: Levels 0–2, MO3, and EX4

Food pathway: LQ3, PU4, and Levels 5–7 (including EC7 and RG7)

Figure 2-1 illustrates the overall IDDSI food and drink classification framework, showing the continuous Level 0–7 scale, corresponding testing methods, and the division into liquid and food pathways.

Figure 2-1. IDDSI Food and Drink Classification and Testing Framework

The framework demonstrates the IDDSI continuum (Levels 0–7), indicating standard testing methods (Flow, Fork, and Spoon Tests) and distinguishing the liquid pathway (Levels 0–2, MO3, EX4) from the food pathway (LQ3, PU4, Levels 5–7 including EC7 and RG7). Twin subtypes (MO3/LQ3, EX4/PU4, EC7/RG7) represent dual-pathway differentiation within the same numeric level.

2.5 Texture Measurements

Texture characteristics were measured using a TA.New Plus texture analyzer (ISENSO, USA). Parameters included hardness, cohesiveness, and adhesiveness, obtained via Texture Profile Analysis (TPA) in double-compression mode.

Testing conditions:
Mode: Compression (Distance-controlled)
Target distance: 3.000 mm
Pre-test and return speed: 0.50 mm/s
Trigger force: 3.000 gf
Each sample was tested three times, and mean values were used as final texture parameters. All measurements were conducted at 25 ± 1 °C to ensure comparability and stability.

2.6 Nutritional Composition Analysis

Nutritional composition per 100 g of each sample was evaluated using Huaxi Hospital Nutrition software, a professional tool with an extensive food database and high calculation precision. Based on food type, weight, and IDDSI level, the software provided detailed estimations of energy, protein, fat, carbohydrate, and dietary fiber contents.

2.7 Statistical Analysis

(1)Descriptive statistics: Texture parameters (hardness, adhesiveness, cohesiveness), moisture content, and nutritional indices (energy, protein, etc.) were summarized by IDDSI level × subtype × food category and presented as median [IQR].
(2)Twin-type comparisons: Differences between twin subtypes (MO3 vs LQ3, EX4 vs PU4, EC7 vs RG7) were assessed using the Mann–Whitney U test with effect size (Cliff's δ) and Benjamini–Hochberg FDR correction ($q \leq 0.05$).
(3)Within-pathway monotonic trends: Level order was treated as an ordinal variable; Spearman's ρ and Kendall's τ_b were computed, and the Theil–Sen slope was used to estimate the average change per level. Pathway trajectories were visualized accordingly.
(4)Intra-level food-category differences: For key levels (L3, L4, L7), Kruskal–Wallis tests compared major food categories. When significant ($p < 0.05$), Dunn–Bonferroni pairwise comparisons followed. Effect size (ε^2) quantified group difference strength, and chi-square tests evaluated category distribution bias across subtypes.
(5)Safety–nutrition balance window: For key nodes (MO3, EX4, PU4, EC7, RG7), P25–P75 (target zone) and P10–P90 (tolerance zone) ranges were calculated for hardness, adhesiveness, moisture, energy, and protein. Confidence intervals (95%) were estimated via bootstrap resampling (n = 1000) to define balance windows.
(6)Software and significance: All analyses were performed in Python 3.9 (pandas, scipy, pingouin, matplotlib) and SPSS 26.0. Significance was determined by two-

tailed $p < 0.05$, with $q \leq 0.05$ after multiple testing correction.

2.8 Data and Ethical Statement

This study involved no human or animal experimentation. All food materials were commercially sourced, and the experimental preparation process complied with the Laboratory Food Safety Code of Practice.

3.Results

3.1 Sample Characteristics and Composition

A total of 585 food samples were included in this study, comprising 301 samples (51.5%) classified under the liquid pathway and 284 samples (48.5%) under the food pathway.According to the International Dysphagia Diet Standardisation Initiative (IDDSI) framework, the liquid pathway covered Levels 0–2 and the subtypes MO3 and EX4, while the food pathway included subtypes LQ3, PU4, and Levels 5–7 (including EC7 and RG7).The sample distribution across IDDSI levels and subtypes was as follows:Level 0 – 110 samples (18.8%), Level 1 – 84 samples (14.4%), Level 2 – 78 samples (13.3%).Within Level 3, MO3 accounted for 24 samples (4.1%) and LQ3 for 57 samples (9.7%).Within Level 4, EX4 included 5 samples (0.9%) and PU4 included 50 samples (8.5%).Higher levels consisted of Level 5 – 63 samples (10.8%), Level 6 – 78 samples (13.3%), Level 7 EC7 – 21 samples (3.6%), and RG7 – 15 samples (2.6%).

The major food-category compositions within each subtype were as follows:Level 0: mainly fruit (32.7%) and vegetable (26.4%).Level 1: dominated by vegetable (39.3%) and fruit (28.6%).Level 2: composed primarily of vegetable (26.9%), fruit (19.2%), and egg (19.2%).Level 3: MO3 was dominated by vegetable (58.3%), while LQ3 was characterized by grains and tubers (35.1%) and vegetable (17.5%).Level 4: EX4 consisted mainly of vegetable (60.0%) and fruit (40.0%); PU4 contained vegetable (36.0%), grains and tubers (26.0%), and egg (24.0%) as primary components.
Level 5: mainly grains and tubers (33.3%), meat (19.0%), and vegetable (19.0%).
Level 6: vegetable (26.9%) and grains and tubers (26.9%) shared similar proportions.
Level 7: EC7 featured fruit (42.9%) and vegetable (28.6%), while RG7 showed a balanced distribution across mixed beans (20.0%), fruit (20.0%), and meat (20.0%).

A summary of the sample composition across IDDSI subtypes is presented in Table 3-1, and the proportional distribution of major food categories within each subtype is illustrated in Figure 3-1.

Table 3-1. Sample composition across IDDSI subtypes (N = 585)

IDDSI level / subtype	n	Percent (%)	Path	Main food category composition (%)
0	110	18.8	Liquid-path	Fruit 32.7%; Vegetable 26.4%; Meat 13.6%; Other 12.7%; Grains and tubers 9.1%; Egg 4.5%; Mixed

				beans 0.9%
1	84	14.4	Liquid-path	Vegetable 39.3%; Fruit 28.6%; Grains and tubers 25.0%; Meat 3.6%; Other 3.6%
2	78	13.3	Liquid-path	Vegetable 26.9%; Fruit 19.2%; Egg 19.2%; Grains and tubers 17.9%; Meat 9.0%; Mixed beans 3.8%; Other 3.8%
MO3	24	4.1	Liquid-path	Vegetable 58.3%; Grains and tubers 16.7%; Fruit 12.5%; Meat 4.2%; Egg 4.2%; Other 4.2%
LQ3	57	9.7	Food-path	Grains and tubers 35.1%; Vegetable 17.5%; Fruit 15.8%; Meat 14.0%; Mixed beans 8.8%; Other 5.3%; Egg 3.5%
EX4	5	0.9	Liquid-path	Vegetable 60.0%; Fruit 40.0%
PU4	50	8.5	Food-path	Vegetable 36.0%; Grains and tubers 26.0%; Egg 24.0%; Meat 8.0%; Fruit 4.0%; Other 2.0%
5	63	10.8	Food-path	Grains and tubers 33.3%; Meat 19.0%; Vegetable 19.0%; Fruit 11.1%; Egg 7.9%; Mixed beans 6.3%; Other 3.2%
6	78	13.3	Food-path	Vegetable 26.9%; Grains and tubers 26.9%; Mixed beans 16.7%; Meat 14.1%; Fruit 9.0%; Egg 3.8%; Other 2.6%
EC7	21	3.6	Food-path	Fruit 42.9%; Vegetable 28.6%; Grains and tubers 28.6%
RG7	15	2.6	Food-path	Mixed beans 20.0%; Fruit 20.0%; Meat 20.0%; Egg 13.3%; Grains and tubers 13.3%; Vegetable 6.7%; Other 6.7%

Figure 3-1. Food category composition (%) within each IDDSI subtype

The figure illustrates the proportional distribution of seven major food categories—grains and tubers, mixed beans, fruit, meat, vegetable, egg, and other (tofu)—within each IDDSI subtype, highlighting distinct ingredient patterns between the liquid and food pathways.

3.2 Comparison between Twin Subtypes / Dual Pathways

This section compares paired subtypes within the same numeric IDDSI levels but belonging to different ingestion pathways, including Level 3 (MO3 vs. LQ3), Level 4 (EX4 vs. PU4), and Level 7 (EC7 vs. RG7). For each pair, texture parameters (hardness, adhesiveness, cohesiveness), hydration indices (water content and water ratio), and nutritional indicators (energy, protein, fat, carbohydrate, and dietary fiber per 100 g) were analyzed. Results are reported as median [IQR] along with *p* values (Mann–Whitney U test), *q* values (Benjamini–Hochberg correction), and effect sizes (Cliff's δ).

Level 3 (MO3 vs. LQ3):MO3 (n = 24) and LQ3 (n = 57) exhibited distinct textural and nutritional profiles. Hardness (118.60 vs. 143.41 N/m², $q > 0.05$) and cohesiveness (0.76 vs. 0.76, $q > 0.05$) were comparable, while adhesiveness tended to be lower in LQ3. Water content was significantly higher in MO3 (96.48 vs. 89.20 g/100 g, $q < 0.05$, $\delta = 0.622$), indicating greater hydration. In contrast, LQ3 showed higher energy (51.00 vs. 11.20 kcal/100 g, $q < 0.05$), protein, fat, and carbohydrate contents, suggesting denser nutritional composition in the food pathway.

Level 4 (EX4 vs. PU4):EX4 (n = 5) and PU4 (n = 50) both represented thickened consistencies but differed in pathway classification. PU4 exhibited higher hardness (160.96 vs. 130.50 N/m², $q > 0.05$) and nutrient density, while EX4 showed significantly greater water content (96.80 vs. 88.45 g/100 g, $q < 0.05$) and water ratio (1.50 vs. 0.50, $q < 0.05$). Nutritionally, PU4 contained higher energy (71.50 vs. 40.00 kcal/100 g, $q < 0.05$), protein, and fat levels, reflecting a trade-off between hydration safety and nutrient concentration.

Level 7 (EC7 vs. RG7):EC7 (n = 21) and RG7 (n = 15) both belong to the food pathway but differ in chewing thresholds. RG7 showed higher hardness (187.47 vs. 150.40 N/m², $q < 0.05$), while adhesiveness and cohesiveness did not differ significantly. Water content was greater in EC7 (89.50 vs. 83.50 g/100 g, $q < 0.05$), facilitating easier bolus formation. Nutritionally, RG7 demonstrated higher energy (71.50 vs. 51.00 kcal/100 g, $q < 0.05$), protein (15.20 vs. 0.90 g/100 g, $q < 0.05$), and fat contents, indicating a denser and more structured composition.

The summarized statistics for all indicators—including median [IQR], *p*, *q*, and Cliff's δ values—are presented in Table 3-2, providing a comprehensive overview of within-level differences.Comparisons of texture parameters are illustrated in Figure 3-2A (Level 3), Figure 3-2B (Level 4), and Figure 3-2C (Level 7), while corresponding nutritional profiles are depicted in Figure 3-2D–F.
Together, these findings highlight a consistent pattern of lower hardness and energy density in liquid-pathway subtypes and higher nutrient concentration in food-pathway counterparts, supporting the concept that "same level ≠ interchangeable."

Table 3-2. Within-level comparison of paired IDDSI subtypes

Level	Indicator	Subtype A Median [IQR]	Subtype B Median [IQR]	*p*-value	q-value	Cliff's δ
Level 3 (MO3 vs LQ3)	Hardness (N/m²)	118.60 [116.76–134.53]	143.41 [103.40–202.37]	＞0.05	＞0.05	–0.183
	Adhesiveness (gf)	–6.00 [–9.10—1.49]	–14.58 [–20.89–0.00]	＞0.05	＞0.05	0.171
	Cohesiveness (unitless)	0.76 [0.71–0.77]	0.76 [0.66–0.94]	＞0.05	＞0.05	–0.137
	Water content (g/100 g)	96.48 [88.90–96.80]	89.20 [85.50–93.92]	＜0.05	＜0.05	0.622
	Water ratio	1.00 [0.50–1.50]	1.00 [0.50–1.50]	＞0.05	＞0.05	–0.032
	Energy (kcal/100 g)	11.20 [9.33–42.00]	51.00 [22.80–71.50]	＜0.05	＜0.05	–0.630
	Protein (g/100 g)	0.93 [0.67–1.13]	1.13 [0.73–4.45]	＜0.05	＜0.05	–0.472
	Fat (g/100 g)	0.15 [0.12–0.16]	0.20 [0.10–0.40]	＜0.05	＜0.05	–0.360
	Carbohydrates (g/100 g)	2.00 [1.50–10.10]	4.96 [2.27–13.10]	＜0.05	＜0.05	–0.328
	Dietary fiber (g/100 g)	0.04 [0.00–0.62]	0.40 [0.00–1.00]	＞0.05	＞0.05	–0.214
Level 4 (EX4 vs PU4)	Hardness (N/m²)	130.50 [127.48–134.04]	160.96 [131.55–214.22]	＞0.05	＞0.05	–0.400
	Adhesiveness (gf)	–17.65 [–22.21––12.60]	–10.40 [–17.00—3.30]	＞0.05	＞0.05	–0.320
	Cohesiveness (unitless)	0.80 [0.74–0.86]	0.80 [0.70–0.89]	＞0.05	＞0.05	–0.080
	Water content (g/100 g)	96.80 [95.00–97.30]	88.45 [82.10–92.30]	＜0.05	＜0.05	0.720
	Water ratio	1.50 [1.50–2.00]	0.50 [0.25–1.00]	＜0.05	＜0.05	0.680
	Energy (kcal/100 g)	40.00 [31.80–47.60]	71.50 [47.30–108.00]	＜0.05	＜0.05	–0.680

	Protein (g/100 g)	1.20 [0.60–1.30]	4.20 [1.30–6.20]	<0.05	<0.05	–0.720
	Fat (g/100 g)	0.20 [0.10–0.30]	0.60 [0.20–2.30]	<0.05	<0.05	–0.640
	Carbohydrates (g/100 g)	9.90 [8.40–12.80]	13.10 [5.10–19.30]	>0.05	>0.05	–0.440
	Dietary fiber (g/100 g)	1.00 [1.00–1.30]	0.30 [0.00–1.20]	>0.05	>0.05	0.280
	Hardness (N/m^2)	150.40 [133.34–169.62]	187.47 [171.22–212.94]	<0.05	<0.05	0.454
	Adhesiveness (gf)	–2.70 [–6.10–0.00]	–3.40 [–9.40–0.00]	>0.05	>0.05	–0.133
	Cohesiveness (unitless)	0.77 [0.72–0.84]	0.80 [0.70–0.87]	>0.05	>0.05	0.308
	Water content (g/100 g)	89.50 [85.20–93.20]	83.50 [79.30–86.10]	<0.05	<0.05	0.543
Level 7 (EC7 vs RG7)	Water ratio	0.50 [0.50–0.50]	0.50 [0.25–0.50]	>0.05	>0.05	–0.200
	Energy (kcal/100 g)	51.00 [31.00–71.50]	71.50 [51.00–91.00]	<0.05	<0.05	–0.543
	Protein (g/100 g)	0.90 [0.40–1.40]	15.20 [1.40–20.20]	<0.05	<0.05	–0.800
	Fat (g/100 g)	0.20 [0.10–0.20]	0.60 [0.20–2.30]	<0.05	<0.05	–0.543
	Carbohydrates (g/100 g)	13.10 [9.90–26.20]	13.10 [3.40–22.00]	>0.05	>0.05	0.114
	Dietary fiber (g/100 g)	1.10 [1.00–2.60]	0.10 [0.00–1.20]	>0.05	>0.05	0.257

Figures for Section 3.2 — Comparison between Twin Subtypes / Dual Pathways

Figure 3-2A. Texture parameter comparison: Level 3 (MO3 vs LQ3)
Boxplots illustrating differences in hardness, adhesiveness, and cohesiveness between Level 3 subtypes.*MO3 (liquid pathway) samples exhibited lower hardness and higher water content, whereas LQ3 (food pathway) showed greater structural density.*

Figure 3-2B. Texture parameter comparison: Level 4 (EX4 vs PU4)

Boxplots comparing texture parameters (hardness, adhesiveness, cohesiveness) of Level 4 subtypes.
PU4 (food pathway) exhibited higher hardness and nutrient density than EX4 (liquid pathway), which maintained higher moisture and lower viscosity.

Figure 3-2C. Texture parameter comparison: Level 7 (EC7 vs RG7)
Boxplots showing the three texture indices across Level 7 subtypes.
RG7 (regular foods) showed significantly higher hardness, while EC7 (easy-to-chew) maintained higher water content and softer texture, supporting safer oral processing.

Figure 3-2D. Nutritional profile comparison: Level 3 (MO3 vs LQ3)
Radar chart comparing five nutritional indicators (energy, protein, fat, carbohydrate, fiber) between Level 3 subtypes.
LQ3 presented markedly higher energy and macronutrient density than MO3.

Figure 3-2E. Nutritional profile comparison: Level 4 (EX4 vs PU4)
Radar chart showing nutritional contrasts between Level 4 subtypes.
PU4 demonstrated a more nutrient-dense composition, whereas EX4 emphasized higher water content and lower energy density.

Figure 3-2F. Nutritional profile comparison: Level 7 (EC7 vs RG7)
Radar chart presenting the nutrient profiles of Level 7 subtypes.
RG7 exhibited higher energy, protein, and fat contents, while EC7 retained higher moisture and easier chewability.

3.3 Ordinal Trends within Each Path

3.3.1 Liquid Path (0 → 1 → 2 → MO3 → EX4)

A progressive, ordinal trend was observed along the liquid pathway, covering Levels 0 to EX4. Table 3-3A summarizes correlation coefficients (Spearman's ρ, Kendall's τ_b), *q* values (Benjamini–Hochberg FDR), and Theil–Sen slopes per level, while the overall monotonic trajectories are visualized in Figure 3-3A.

In terms of texture, hardness increased significantly with ascending levels ($\rho = 0.686$, $q < 0.05$), with a Theil–Sen slope of approximately +9.87 N/m^2 per level.Adhesiveness showed a strong negative correlation ($\rho = –0.627$, $q < 0.05$), while cohesiveness also declined ($\rho = –0.628$, $q < 0.05$).For hydration-related indicators, water content per 100 g exhibited a decreasing trend with increasing level ($\rho = –0.192$, $q < 0.05$), while the water ratio dropped even more markedly ($\rho = –0.385$, $q < 0.05$; Theil–Sen ≈ –0.33 per level). Median water content decreased from 84.35 g/100 g at Level 0 to approximately 150.79 g/100 g at EX4.Regarding nutritional indicators, a mild but consistent positive trend was detected for energy density ($\rho = 0.159$, $q < 0.05$;

+0.50 kcal/100 g per level) and macronutrients. Protein (ρ = 0.291, $q < 0.05$; +0.06 g/100 g per level), fat (ρ = 0.243, $q < 0.05$; +0.03 g/100 g per level), and carbohydrate (ρ = 0.162, $q < 0.05$; +0.35 g/100 g per level) all increased progressively, whereas dietary fiber showed no significant association (ρ = 0.067, $q > 0.05$). Median energy rose from approximately 11.20 kcal/100 g at Level 0 to 40.00 kcal/100 g at EX4, and protein increased from 0.93 g/100 g to 1.20 g/100 g .

Overall, within the liquid path, hardness demonstrated a strong upward monotonic trend ($q \leq 0.05$), adhesiveness decreased markedly, and hydration indices consistently declined. In contrast, energy, protein, fat, and carbohydrate contents increased with higher levels ($q \leq 0.05$), while dietary fiber remained largely unchanged ($q > 0.05$).

Table 3-3A. Spearman's ρ, *q* values, Kendall's τ_b, and Theil–Sen slopes for indicators along the Liquid Path (0 → 1 → 2 → MO3 → EX4).

Metric	Spearman ρ	*q* (BH-FDR)	Kendall τ_b	Theil–Sen slope / level	Start → End (median [IQR])
Hardness	0.686	<0.05	0.552	+9.87	0: 84.35 [82.09–88.23] → EX4: 150.79 [101.73–…]
Adhesiveness	–0.627	<0.05	–0.544	~0 (downward)	0: –6.00 [–9.10—1.49] → EX4: –17.65 [–22.21—12.60]
Cohesiveness	–0.628	<0.05	–0.494	–0.111	0: 0.76 [0.71–0.77] → EX4: 0.80 [0.74–0.86]
Water content	–0.192	<0.05	–0.150	–0.70	0: 84.35 [82.09–88.23] → EX4: 150.79 [101.73–197.77]
Water ratio	–0.385	<0.05	–0.321	–0.33	0: 1.50 [1.50–2.00] → EX4: 0.50 [0.25–1.00]
Energy (kcal/100 g)	0.159	<0.05	0.122	+0.50	0: 11.20 [9.33–42.00] → EX4: 40.00 [31.80–47.60]
Protein (g/100 g)	0.291	<0.05	0.244	+0.06	0: 0.93 [0.67–1.13] → EX4: 1.20 [0.60–1.30]

Fat (g/100 g)	0.243	<0.05	0.195	+0.03	0: 0.15 [0.12–0.16] → EX4: 0.20 [0.10–0.30]
Carbohydrate (g/100g)	0.162	<0.05	0.127	+0.35	0: 2.00 [1.50–10.10] → EX4: 9.90 [8.40–12.80]
Dietary fiber (g/100g)	0.067	>0.05	0.065	+0.02	0: 0.04 [0.00–0.62] → EX4: 1.00 [1.00–1.30]

Significance judged at $q \le 0.05$. EX4 water content >100 g/100 g suggests inclusion of free water during measurement; data reported as-is.

Figure 3-3A. Ordinal trend along the Liquid Path

(Levels 0 → 1 → 2 → MO3 → EX4)

3.3.2 Food Path (LQ3 → PU4 → 5 → 6 → EC7 → RG7)

Along the food pathway, covering Levels LQ3 to RG7, texture and nutritional characteristics exhibited distinct ordinal trends (Table 3-3B; Figure 3-3B). Hardness increased significantly with ascending levels (Spearman $\rho = 0.808$, $q < 0.05$), with a Theil–Sen slope of approximately +198 N/m^2 per level. The median hardness rose from 143.41 N/m^2 at LQ3 to 1242.23 N/m^2 at RG7. Adhesiveness ($\rho = –0.048$, $q > 0.05$) and cohesiveness ($\rho = –0.055$, $q > 0.05$) showed no significant monotonic trends.Water content decreased significantly with level advancement ($\rho = –0.277$, $q < 0.05$; Theil–Sen ≈ –1.94 g/100 g per level), dropping from 89.20 g/100 g at LQ3 to 75.20 g/100 g at RG7. Similarly, the water ratio exhibited a pronounced decline ($\rho = –0.500$, $q < 0.05$; –0.25 per level), from 1.00 at LQ3 to 0.00 [0.00–0.00] at RG7.Energy content increased significantly with level ($\rho = 0.223$, $q < 0.05$; +0.66 kcal/100 g per level), rising from 51.00 kcal/100 g at LQ3 to 106.00 kcal/100 g at RG7. Carbohydrates ($\rho = 0.267$, $q < 0.05$; +1.05 g/100 g per level) and dietary fiber ($\rho = 0.212$, $q < 0.05$; +0.02 g/100 g per level) also exhibited significant upward trends. Protein ($\rho = 0.109$, $q = 0.085$) and fat ($\rho = 0.119$, $q = 0.063$) showed weak positive associations that did not reach statistical significance after FDR correction.Nevertheless, endpoint comparisons revealed substantial increases: protein rose from 1.13 g/100 g in LQ3 to 15.20 g/100 g in RG7, and fat increased from 0.20

g/100 g to 0.60 g/100 g.
Within the food path, hardness showed a strong positive monotonic trend ($q \leq 0.05$), while water content and water ratio decreased significantly ($q \leq 0.05$). Energy, carbohydrate, and dietary fiber content increased progressively. Adhesiveness and cohesiveness did not vary significantly across levels ($q > 0.05$).

Table 3-3B. Spearman's ρ, *q* values, Kendall's τ_b, and Theil–Sen slopes for indicators along the Food Path (LQ3 → PU4 → 5 → 6 → EC7 → RG7).

Metric	Spearman ρ	q (BH-FDR)	Kendall τ_b	Theil–Sen slope / level	Start → End (median [IQR])
Hardness	0.808	⩽0.05	0.659	+198.11	LQ3: 143.41 [103.40–202.37] → RG7: 1242.23 [1024.63–1418.22]
Adhesiveness	–0.048	>0.05	–0.025	–0.094	LQ3: –14.58 [–20.89–0.00] → RG7: –0.62 [–25.62–0.00]
Cohesiveness	–0.055	>0.05	–0.044	–0.0064	LQ3: 0.76 [0.66–0.94] → RG7: 0.78 [0.77–0.83]
Water content	–0.277	⩽0.05	–0.206	–1.94	LQ3: 89.20 [85.50–93.92] → RG7: 75.20 [51.50–78.10]
Water ratio	–0.500	⩽0.05	–0.413	–0.25	LQ3: 1.00 [0.50–1.50] → RG7: 0.00 [0.00–0.00]
Energy (kcal/100 g)	0.223	⩽0.05	0.167	+0.66	LQ3: 51.00 [22.80–71.50] → RG7: 106.00 [93.40–119.00]
Protein (g/100 g)	0.109	⩽0.05	0.083	+0.125	LQ3: 1.13 [0.73–4.45] → RG7: 15.20 [1.40–20.20]
Fat (g/100 g)	0.119	⩽0.05	0.087	+0.010	LQ3: 0.20 [0.10–0.40] → RG7: 0.60 [0.20–2.30]
Carbohydrate (g/100g)	0.267	⩽0.05	0.200	+1.05	LQ3: 4.96 [2.27–13.10] → RG7:

					13.10 [3.40–22.00]
Dietary fiber (g/100g)	0.212	⩽0.05	0.168	+0.02	LQ3: 0.40 [0.00–1.00] → RG7: 0.10 [0.00–1.20]

Significance judged at $q \leq 0.05$.

Figure 3-3B. Ordinal trend along the Food Path

(LQ3 → PU4 → 5 → 6 → EC7 → RG7)

3.4 Intra-level Variability by Food Category

Level 3 (MO3 + LQ3 combined as "IDDSI Level 3")： Level 3 included 81 samples: grains and tubers (n = 24), vegetables (n = 24), fruit (n = 12), meat (n = 6), mixed beans (n = 6), other: tofu (n = 6), and egg (n = 3).Kruskal–Wallis tests showed significant differences across food categories for all indicators, with all results remaining significant after FDR correction ($q \leq 0.05$): hardness (H = 35.77), adhesiveness (H = 38.99), cohesiveness (H = 33.65), water content (H = 52.02), energy (H = 54.96), and protein (H = 55.23). Effect sizes (ε^2) ranged from 0.37 to 0.67.Median hardness ranged from 108 N/m^2 in grains and tubers to 214 N/m^2 in other: tofu; egg showed 179.39 N/m^2. Adhesiveness varied across categories (e.g. fruit: –27.34 gf; grains and tubers: –1.33 gf; meat: 0.00 gf). Water content also differed (mixed beans: 67.72 g/100 g ; grains and tubers: 92.17 g/100 g ; vegetables: 89.20 g/100 g).Energy and protein content were higher in egg, meat, and mixed beans than in fruit, vegetables, and grains and tubers (e.g. egg: 109.33 kcal/100 g and 6.84 g/100 g protein; fruit: 46.50 kcal/100 g and 0.50 g/100 g protein). A subtype × category χ^2 test indicated a non-random distribution of food categories between MO3 and LQ3.

Level 4 (EX4 + PU4 combined as "IDDSI Level 4")： Level 4 included 55 samples: vegetables (n = 21), grains and tubers (n = 13), egg (n = 12), fruit (n = 3), meat (n = 3), and mixed beans (n = 3).All six indicators differed significantly across food categories (all $q \leq 0.001$): hardness (H = 24.74), adhesiveness (H = 22.88), cohesiveness (H = 22.47), water content (H = 31.12), energy (H = 38.22), and protein (H = 36.30). Effect sizes (ε^2) ranged from 0.36 to 0.68.Median hardness in egg (367.96 N/m^2) exceeded fruit (98.36 N/m^2) and meat (124.07 N/m^2). Water content differed between categories (vegetables: 95.20 g/100 g; egg: 78.17 g/100 g; mixed beans: 75.16 g/100 g). Energy and protein were higher in egg and mixed beans than in vegetables and grains and tubers (egg: 147.60 kcal/100 g and 6.84 g/100 g protein;

vegetables: 14.00 kcal/100 g and 1.10 g/100 g protein). A χ^2 test showed that food categories were not evenly distributed between EX4 and PU4.

Level 7 (EC7 + RG7 combined as "IDDSI Level 7"): Level 7 included 30 samples: fruit (n = 12), grains and tubers (n = 9), vegetables (n = 6), egg (n = 3), meat (n = 3), and mixed beans (n = 3).Kruskal–Wallis tests were significant for all indicators ($q \leq 0.05$): hardness (H = 27.82, $\varepsilon^2 = 0.76$), adhesiveness (H = 14.75, $\varepsilon^2 = 0.32$), cohesiveness (H = 22.69, $\varepsilon^2 = 0.59$), water content (H = 28.33, $\varepsilon^2 = 0.78$), energy (H = 28.33, $\varepsilon^2 = 0.78$), and protein (H = 27.68, $\varepsilon^2 = 0.76$).Median hardness spanned widely across categories (egg: 744.05 N/m² [718.39–788.63]; meat: 1050.77 N/m² ; grains and tubers: 1927.13 N/m² [1665.60–2723.51]; fruit: 6699.57 N/m²). Water content also differed (vegetables: 92.20 g/100 g [89.20–95.20]; fruit: 86.00 g/100 g; mixed beans: 12.60 g/100 g).Energy and protein content were highest in egg and mixed beans (egg: 328.00 kcal/100 g and 15.20 g/100 g protein; mixed beans: 324.00 kcal/100 g and 20.20 g/100 g protein) and lower in fruit (52.00 kcal/100 g and 0.50 g/100 g protein). A χ^2 test indicated non-random distribution of food categories between EC7 and RG7.

Table 3-4. Within-level variability by food category across IDDSI Level 3, Level 4, and Level 7, including sample size per category, median [IQR] for hardness, adhesiveness, cohesiveness, water content, energy, and protein, and Kruskal–Wallis results (ε^2 and FDR-adjusted *q* values).

Table 3-4A. Within-level variability by food category in IDDSI Level 3 (MO3 + LQ3 combined).

Food category	n	Hardness (N/m²) median [IQR]	Adhesiveness (gf) median [IQR]	Cohesiveness median [IQR]	Water content (g/100 g) median [IQR]	Energy (kcal/100 g) median [IQR]	Protein (g/100 g) median [IQR]
Egg	3	179.39 [159.03–211.92]	–15.59 [–17.00—15.07]	0.71 [0.68–0.71]	83.83 [83.83–83.83]	109.33 [109.33–109.33]	6.84 [5.83–8.23]
Fruit	12	156.03 [132.85–225.52]	–27.34 [–32.95—18.85]	0.75 [0.73–0.76]	87.50 [86.05–88.90]	46.50 [42.00–51.50]	0.50 [0.50–0.50]
Grains and tubers	24	108.00 [96.10–127.73]	–1.33 [–8.95–0.00]	0.87 [0.77–1.00]	92.17 [62.20–	30.57 [26.41–146.23]	0.95 [0.52–1.40]

					93.36]		
Meat	6	109.75 [103.41–114.70]	0.00 [–0.08–0.00]	0.87 [0.81–0.95]	86.15 [85.50–86.80]	61.50 [51.50–71.50]	5.05 [5.05–5.05]
Mixed beans	6	161.60 [116.89–207.31]	–7.48 [–15.70—0.23]	0.67 [0.57–0.77]	67.72 [56.15–79.30]	122.91 [81.33–164.50]	4.45 [4.45–4.45]
Other：tofu	6	214.48 [176.57–249.73]	–20.48 [–22.11—16.95]	0.61 [0.60–0.63]	89.20 [89.20–89.20]	56.00 [56.00–56.00]	2.80 [2.80–2.80]
Vegetable	24	138.11 [118.38–162.96]	–6.46 [–14.15—1.03]	0.76 [0.74–0.90]	89.20 [86.50–91.95]	56.00 [56.00–56.00]	1.10 [1.00–1.40]

Kruskal–Wallis, FDR-adjusted $q \leq 0.05$.

Table 3-4B. Within-level variability by food category in IDDSI Level 4 (EX4 + PU4 combined).

Food category	n	Hardness (N/m²) median [IQR]	Adhesiveness (gf) median [IQR]	Cohesiveness median [IQR]	Water content (g/100 g) median [IQR]	Energy (kcal/100 g) median [IQR]	Protein (g/100 g) median [IQR]
Egg	12	367.96 [254.92–605.40]	–12.65 [–17.07––5.88]	0.69 [0.56–0.75]	78.17 [73.73–81.41]	147.60 [125.73–177.67]	6.84 [5.83–8.23]
Fruit	3	98.36 [94.99–100.04]	–1.77 [–1.94—0.91]	0.79 [0.79–0.86]	83.87 [83.87–83.87]	62.27 [62.27–62.27]	0.93 [0.93–0.93]
Grains and tubers	13	212.78 [176.16–340.47]	–31.37 [–45.39––19.66]	0.69 [0.66–0.78]	95.10 [90.13–95.10]	19.67 [19.67–39.89]	0.47 [0.43–1.38]
Meat	3	124.07 [120.32–125.33]	0.00 [0.00–0.00]	0.90 [0.89–0.90]	93.80 [93.80–93.80]	26.50 [26.50–26.50]	5.05 [5.05–5.05]
Mixed	3	299.09	–26.94	0.51	75.16	97.60	1.80

beans		[285.98–302.44]	[–27.45––24.73]	[0.50–0.51]	[75.16–75.16]	[97.60–97.60]	[1.80–1.80]
Vegetable	21	183.17 [169.38–297.13]	–19.02 [–53.13––10.13]	0.70 [0.68–0.79]	95.20 [94.60–95.80]	14.00 [13.50–20.60]	1.10 [0.90–1.40]

Kruskal–Wallis, FDR-adjusted $q \leq 0.05$.

Table 3-4C. Within-level variability by food category in IDDSI Level 7 (EC7 + RG7 combined).

Food category	n	Hardness (N/m²) median [IQR]	Adhesiveness (gf) median [IQR]	Cohesiveness median [IQR]	Water content (g/100 g) median [IQR]	Energy (kcal/100 g) median [IQR]	Protein (g/100 g) median [IQR]
Egg	3	744.05 [718.39–788.63]	–0.42 [–0.83—0.41]	0.91 [0.90–0.93]	51.50 [51.50–51.50]	328.00 [328.00–328.00]	15.20 [15.20–15.20]
Fruit	12	6699.57 [5299.72–9671.19]	–20.44 [–36.53––2.25]	0.87 [0.83–0.89]	86.00 [83.38–86.80]	52.00 [48.75–63.10]	0.50 [0.38–0.80]
Grains and tubers	9	1927.13 [1665.60–2723.51]	–16.06 [–25.42––10.88]	0.84 [0.80–0.86]	72.60 [5.60–85.30]	108.00 [59.00–362.00]	1.40 [1.30–8.40]
Meat	3	1050.77 [1013.99–1055.59]	–0.22 [–0.28—0.11]	0.77 [0.77–0.77]	75.20 [75.20–75.20]	106.00 [106.00–106.00]	20.20 [20.20–20.20]
Mixed beans	3	1242.23 [1218.67–1471.55]	–0.54 [–0.58—0.34]	0.51 [0.50–0.53]	12.60 [12.60–12.60]	324.00 [324.00–324.00]	20.20 [20.20–20.20]
Vegetable	6	1649.70 [772.54–2816.04]	–5.99 [–21.34—0.43]	0.70 [0.56–0.79]	92.20 [89.20–95.20]	28.10 [15.00–41.20]	0.95 [0.90–1.00]

Kruskal–Wallis, FDR-adjusted $q \leq 0.05$.

3.5 Safety–Nutrition Trade-off Windows

Table 3-5 summarizes the empirical distribution windows of key indicators—hardness, adhesiveness, water content, energy, and protein—across representative IDDSI subtypes (MO3, EX4, PU4, EC7, RG7).

For each subtype, the interquartile range (P25–P75) represents the target operational band, and the P10–P90 interval represents the release tolerance range.

In MO3, hardness ranged from 116.76–134.53 N/m² (P25–P75), adhesiveness from –9.10 to –1.49 gf, and water content from 88.90–96.80 g/100 g. Energy and protein densities were 9.33–42.00 kcal/100 g and 0.67–1.13 g/100 g, respectively.
EX4 showed hardness of 101.73–159.82 N/m², adhesiveness –18.27 to –2.10 gf, and water content 88.90–94.80 g/100 g; energy ranged from 14.00–62.27 kcal/100 g, and protein 0.93–1.30 g/100 g.
PU4 presented hardness of 176.67–350.30 N/m², adhesiveness –43.44 to –9.20 gf, water content 75.16–95.10 g/100 g, energy 15.33–109.33 kcal/100 g, and protein 0.91–5.05 g/100 g.
EC7 showed hardness of 2438.74–5400.02 N/m², adhesiveness –24.63 to –1.14 gf, and water content 72.60–89.20 g/100 g. Energy ranged 41.20–108.00 kcal/100 g, and protein 0.40–1.40 g/100 g.
RG7 exhibited hardness of 1013.99–1683.24 N/m², adhesiveness –25.62 to –0.37 gf, water content 51.50–75.80 g/100 g, energy 93.40–324.00 kcal/100 g, and protein 1.40–20.20 g/100 g.

Overall, the data indicate a gradual increase in hardness and nutritional density, accompanied by a decline in hydration indicators, from liquid-path (MO3–EX4) to food-path (PU4–RG7) subtypes.

Table 3-5. Suggested operational windows for key IDDSI subtypes (*P25–P75 = target band; P10–P90 = release tolerance band.*)

Subtype (n)	Parameter	P25–P75 (Target range)	P10–P90 (Release range)
MO3 (n=24)	Hardness (N/m²)	116.76–134.53	114.27–151.71
	Adhesiveness (gf)	-9.10 – -1.49	-11.98 – -0.35
	Water content (g/100 g)	88.90–96.80	79.30–97.40
	Energy (kcal/100 g)	9.33–42.00	7.00–81.33
	Protein (g/100 g)	0.67–1.13	0.60–4.60
EX4 (n=5)	Hardness (N/m²)	101.73–159.82	95.67–164.35
	Adhesiveness (gf)	-18.27 – -2.10	-18.72 – -1.90
	Water content (g/100 g)	88.90–94.80	88.90–95.20
	Energy (kcal/100 g)	14.00–62.27	14.00–62.27
	Protein (g/100 g)	0.93–1.30	0.93–1.30
PU4 (n=50)	Hardness (N/m²)	176.67–350.30	160.33–568.84
	Adhesiveness (gf)	-43.44 – -9.20	-55.63 – -2.19
	Water content (g/100 g)	75.16–95.10	75.16–95.20
	Energy (kcal/100	15.33–109.33	13.50–181.00

	g)		
	Protein (g/100 g)	0.91–5.05	0.47–7.60
	Hardness (N/m²)	2438.74–5400.02	860.65–12042.51
	Adhesiveness (gf)	-24.63 – -1.14	-28.94 – -0.30
EC7 (n=21)	Water content (g/100 g)	72.60–89.20	5.60–95.20
	Energy (kcal/100 g)	41.20–108.00	15.00–362.00
	Protein (g/100 g)	0.40–1.40	0.30–8.40
	Hardness (N/m²)	1013.99–1683.24	779.72–7376.46
	Adhesiveness (gf)	-25.62 – -0.37	-38.77 – -0.18
RG7 (n=15)	Water content (g/100 g)	51.50–75.80	12.60–85.30
	Energy (kcal/100 g)	93.40–324.00	59.00–328.00
	Protein (g/100 g)	1.40–20.20	1.30–20.20

4. Discussion

4.1 Mechanistic Explanation and Constitutive Control of Intra-Level Differentiation

This study systematically compared dual pathways/subtypes (MO3 vs. LQ3, EX4 vs. PU4, EC7 vs. RG7) within IDDSI Levels 3, 4, and 7. The findings indicate that liquid path subtypes (MO3, EX4) generally exhibit lower hardness, less adhesive burden, and higher water content compared to food path subtypes (LQ3, PU4), while food path subtypes display significantly higher energy and protein content ($p < 0.05$, FDR-BH corrected $q \leq 0.05$). At Level 7, EC7 demonstrates lower hardness and adhesiveness compared to RG7, with negligible differences in cohesiveness[6]. These results remain stable when analyzed by food categories, suggesting that “same level ≠ interchangeable” is not due to sample composition bias but reflects differences in swallowing goals and test thresholds [5, 7]. The liquid path focuses on controlled flow/reducing aspiration, while the food path emphasizes agglomeration/crushing and sliding [10, 14]. EC7 in Level 7 reduces the bite and particle size threshold, making it more suitable for individuals with insufficient oral strength and fatigue[6]. These findings align with the IDDSI’s path-based design, providing objective support against direct level-based swapping across pathways in clinical practice[5, 7, 13].

4.2 Ordinal Trends within Pathways and Functional Trajectories

The study identified a continuous “thickening-hardening-dehydrating” trajectory along the Liquid path (0 → 4), where hardness and adhesiveness increase, and cohesiveness and water content decrease with increasing levels. Nutritional parameters (calories, protein, fat, carbohydrates) show weak-to-moderate positive

correlations ($q \leq 0.05$), suggesting that nutritional density depends more on formula enhancement rather than consistency itself[15, 16]. In contrast, the Food path (3 → 7) follows a "structural-hardening-dehydrating" trajectory, where hardness increases sharply, and water content decreases significantly ($q \leq 0.05$). However, adhesiveness and cohesiveness show unstable monotonic relationships, reflecting the complex influence of particle size, breakage/crushing, pore structure, and surface characteristics on oral-pharyngeal transport in levels 5–7[9, 11, 12, 17]. These two trajectories align with previous findings on the trade-off between thickening, aspiration, and residue, and the role of particle size in swallowing triggers and shear stress [12, 14]. The study thus provides coherent and interpretable evidence for the "liquid difficulty/solid tolerable" and "drinking smoothly/solid difficulty" phenotypes[4].

4.3 Clinical Application Transformation: Pathway-Priority Prescription and Substitution Boundaries

Our results suggest that for liquid swallowing difficulty patients (e.g., prone to choking, delayed triggers, need for hydration/medication), gradual progression within the Liquid path (MO3 → EX4) is recommended. This pathway leverages low hardness/low adhesiveness and relatively higher water content to reduce aspiration and residue, while formula enhancements (protein/fat/carbohydrates) can compensate for nutritional needs[15]. Substituting LQ3/PU4 across pathways is not recommended, as this could lead to conflicting effects on hardness, adhesiveness, water content, and nutrition, increasing post-swallow residue or compliance risks (as confirmed in sections 3.2 and 3.5, with significant and systematized differences between EX4/PU4 and MO3/LQ3, $q \leq 0.05$) [7, 13].

For solid swallowing difficulty patients (e.g., chewing/aggregation limitations, but able to drink), a Food path progression is recommended: LQ3 → PU4 → EC7 → RG7. The key is managing particle size, crushability, and water content: within the Food path, hardness increases, and water content decreases with level progression ($q \leq 0.05$). Care should be taken to avoid drastic transitions in hardness that could cause fatigue or residue [17]; EC7, in Level 7, lowers bite/crumble thresholds, improving compliance and safety[6]. For individuals with morning/weak-phase liquid difficulty but solid difficulty during meals, the “within-path switch + daily distribution” approach is advisable: use MO3/EX4 for drinking/medication, and PU4/EC7 for meals. Avoid cross-path substitutions such as EX4 ↔ PU4 or MO3 ↔ LQ3, as this may compromise the balance of “safety-hydration-nutrition”[7, 13]. The IDDSI bedside test standard should be used as the minimum threshold[5], and TPA indicators (hardness, adhesiveness, cohesiveness) + water ratio/content + calories/protein should be used as parallel KPIs[10, 13]. Adjust only one dimension at a time (e.g., particle size/water content first, then nutritional enhancement)[15], and incorporate post-meal residue, drinking volume, and fatigue scores for closed-loop evaluation and adjustments [3].

4.4 Safety-Nutrition Trade-Off Windows: Translating Data into Clinical Quality Control

In order to translate the results into actionable quality control indicators, this study identified the operational windows for key IDDSI subtypes (MO3, EX4, PU4, EC7, RG7) based on the empirical distributions of hardness, adhesiveness, water content, energy density, and protein density. For each subtype, the 25th–75th percentile range (target range) and the 10th–90th percentile range (release tolerance range) were calculated. Table 3-5 summarizes these operational windows, providing clear targets for clinical prescription and substitution boundaries.

The data reveal that Liquid-path subtypes (MO3, EX4) tend to fall within a "low hardness, high water, moderate-low energy/protein" window, while Food-path and chewing-end subtypes (PU4, EC7, RG7) progressively enter a "higher hardness, lower water, higher energy/protein" window[13, 15]. Notably, even within the same numeric IDDSI level (e.g., EX4 vs. PU4 at Level 4, EC7 vs. RG7 at Level 7), the target ranges for these subtypes do not overlap, indicating that cross-path substitution is not equivalent[13]. This reinforces the necessity for pathway-priority approaches in clinical practice, especially for patients with liquid or solid swallowing difficulties[7]. The operational windows presented here offer a minimum standard for clinical practitioners to guide food texture adjustments and recipe formulations, ensuring a balance between safety, hydration, and nutrition [10, 13, 16].

5. Conclusion

This study, centered around the concept of "safety-nutrition balance", systematically quantified the coupling relationships between texture (hardness, adhesiveness, cohesiveness), water content, and nutritional indicators (energy, protein) within the IDDSI continuum (Levels 0–7) using a sample of commonly preferred foods by elderly individuals in China (N=585). The study also validated the objective differences in the "dual pathways/dual subtypes" model across Levels 3, 4, and 7. The results indicate that same numeric levels are not interchangeable. At Levels 3 (MO3 vs. LQ3), 4 (EX4 vs. PU4), and 7 (EC7 vs. RG7), liquid path subtypes generally show lower hardness and adhesiveness, higher water content, while food path subtypes have a distinct advantage in energy density and protein content ($p < 0.05$, FDR-BH corrected $q \leq 0.05$). This directional difference remains statistically significant even after stratifying by food categories (most $q \leq 0.05$), suggesting that these differences arise from the goals of swallowing control and testing thresholds, rather than sample composition bias.

Further path sequence analysis revealed two clear functional trajectories: The Liquid-path ($0 \rightarrow 4$) follows a "thickening-hardening-dehydration" continuum, where hardness and adhesiveness increase, and water content decreases with level progression. Nutritional density increases mainly due to formula fortification, rather than viscosity changes. In contrast, the Food-path ($3 \rightarrow 7$) exhibits a

"structural-hardening-dehydration" trend, with hardness increasing and water content decreasing significantly ($q \leq 0.05$). However, adhesiveness and cohesiveness show unstable monotonic relationships, influenced by multiple factors such as particle size, crushability, and pore structure. Together, these two paths provide an interpretable texture-function continuum for the "liquid difficulty/solid tolerable" and "solid difficulty/liquid smooth" clinical phenotypes.

Based on these findings, clinical and formulation practices should prioritize pathway determination and avoid level-based cross-substitution: For patients with liquid swallowing difficulties (e.g., prone to choking, delayed triggers, need for hydration/medication), gradual progression within the Liquid-path (MO3 → EX4) is recommended, balancing low hardness, high water content, and moderate formula fortification for safety and nutrition. For patients with solid swallowing difficulties (e.g., limited chewing/aggregation but able to drink), gradual progression along the Food-path (LQ3 → PU4 → EC7 → RG7) is advised, controlling the hardness gradient and water reduction rate to prevent chewing fatigue and residue. Cross-path substitutions (e.g., EX4 ↔ PU4, MO3 ↔ LQ3) should be avoided to prevent conflicting interactions in hardness, adhesiveness, and nutritional density, which could increase post-swallow residue or noncompliance.EC7, within Level 7, offers a safer eating option for elderly and individuals with limited chewing strength by lowering particle strength and bite thresholds. In terms of quality control, this study established safety-nutrition balance windows based on percentile method: P25–P75 as the target range and P10–P90 as the release tolerance range, forming operational benchmarks for key subtypes such as MO3, EX4, PU4, EC7, and RG7. It is recommended to use IDDSI bedside testing as the entry threshold, incorporating TPA (Texture Profile Analysis) indicators (hardness/adhesiveness/cohesiveness) along with water ratio, energy, and protein as joint KPIs. This approach can be integrated into a standard operating procedure (SOP) spanning from kitchen to ward to rehabilitation, ensuring a quantifiable connection between food selection, processing techniques, and clinical prescriptions.

In summary, this study is the first to translate the IDDSI numeric level framework into a pathway-based texture-water-nutrition continuum and prescription boundary system. It provides traceable evidence for optimizing the safety, hydration, nutritional adequacy, and patient compliance of dysphagia diets in the Chinese context. Future research should involve multi-center prospective studies, combining VFSS/FEES swallowing imaging and clinical outcome data, to further establish quantitative mappings between key physical parameters, swallowing safety, aspiration risk, and energy sufficiency. This will advance the prescription, standardization, and intelligent application of swallowing foods.

References

[1]Zhang, M.; Li, C.; Dou, Z.; He, C.; Lin, Q.; Yan, T.; Yu, C.; Song, P.; Li, Y.; Wang, M.; et al. Prevalence of Dysphagia in China: An Epidemiological Survey of 5943 Participants. *Dysphagia* 2021, *36*, 628–639. doi:10.1007/s00455-020-10173-1.

[2]Cichero, J.A.Y.; Altman, K.W.; Garcia, A.; Hulme, A.; Intraligi, M.; Kassis, C.A.; Laursen, T.S.; Macleod, H.; Merl, H.; Miyamoto, Y.; et al. Head and Neck Cancer and Dysphagia: An International Dysphagia Diet Standardisation Initiative (IDDSI) Perspective. *J Clin Med* 2022, *11*, 6010. doi:10.3390/jcm11206010.

[3]Zhang, J.; Wang, C.; Wang, Y.; Li, Y.; Wang, J.; Li, S.; Liu, C.; Li, Y. Dysphagia Management and Outcomes in Elderly Stroke Patients with Malnutrition Risk: Results from Chinese Stroke Center Alliance. *Clin Interv Aging* 2022, *17*, 295–308. doi:10.2147/CIA.S355047.

[4]Sun, J.; Wang, R.; Chen, S.; Li, Q.; Li, X.; Chen, Z.; Liu, Y.; Wang, M.; Gao, T.; Chen, J.; et al. Dysphagia, Aspiration Pneumonia, Malnutrition, Frailty, and Reduced Quality of Life: A Literature Review. *Foods* 2024, *13*, 1629. doi:10.3390/foods13111629.

[5] Cichero, J.A.Y.; Lam, P.; Steele, C.M.; Hanson, B.; Chen, J.; Dantas, R.O.; Duivestein, J.; Kayashita, J.; Lecko, C.; Logemann, J.A.; et al. Development of International Dysphagia Diet Standardisation Initiative (IDDSI) Guidelines. *Nutrients* 2017, *9*, 794. doi:10.3390/nu9070794.

[6]Cichero, J.A.Y.; Steele, C.M.; Hanson, B.; Lam, P.; Chen, J.; Kayashita, J. Regular 7 Easy to Chew: An IDDSI Subcategory. *Dysphagia* 2019, *34*, 584–587. doi:10.1007/s00455-019-10022-7.

[7]Steele, C.M.; Namasivayam, A.M.; Macleod, H.; Nogueira, E.; Steele, L. International Dysphagia Diet Standardisation Initiative (IDDSI) implementation: Challenges and opportunities for researchers and clinicians. *Dysphagia* 2020, *35*, 703–709. doi:10.1007/s00455-020-10118-8.

[8]Riquelme, N.; Rojas, M.L.; Tapia, R.D.; Ceballos, J. Texture-Modified Diets for the Management of Dysphagia: A Comprehensive Review on Rheological and Textural Properties, Quality and Sensory Aspects. *Foods* 2023, *12*, 1980. doi:10.3390/foods12101980.

[9]Hadde, E.; Chen, J. A Critical Review on Rheology and Texture of Fluid and Semi-Solid Foods for Dysphagia Management. *Foods* 2022, *11*, 1629. doi:10.3390/foods11111629.

[10]Zhou, W.; Li, X.; Chen, J.; Liu, Y. Instrumental Texture Assessment of IDDSI Texture Levels for Dysphagia Management. Part 2: Texture Modified Foods. *J Texture Stud* 2022, *53,* 821–834. doi:10.1111/jtxs.12701.

[11]Rangarajan, V.; Orellana, J.; Al-Ghorbani, M.; Chen, J. Inter- and Intra-Level Heterogeneity of Texture-Modified Foods based on Starch, Protein, and Gum Systems. *Food Hydrocoll* 2024, *146,* 109267. doi:10.1016/j.foodhyd.2023.109267.

[12] Li, Q.; Li, X.; Chen, Z.; Liu, Y.; Wang, M.; Gao, T.; Chen, J.; Mao, L. Effects of different preparation conditions on microstructure, rheological and tribological properties of food gels and their swallowing performance in a rat model. *J Food Eng* 2022, *318,* 110878. doi:10.1016/j.foodeng.2021.110878.

[13]Wong, L.N.Y.; Chan, M.W.L.; Ng, H.K.; Yuen, P.S.F. Quantitative Textural and Rheological Data on Different Levels of Texture-Modified Food and Thickened Liquids Classified Using the International Dysphagia Diet Standardisation Initiative (IDDSI) Guideline. *Foods* 2023, *12,* 3765. doi:10.3390/foods12203765.

[14] Chen, J.; Sun, J.; Wang, Y.; Mao, L. In vivo evaluation of swallowing safety for texture-modified foods: Insights from animal and human studies. *J Food Eng* 2024, *369*, 111985. doi:10.1016/j.foodeng.2024.111985.

[15]Zhou, W.; Li, X.; Chen, J.; Liu, Y. Designing High-Energy and High-Protein Density Food Gels for Dysphagia Management: A Review on Ingredient Interactions and Texture Control. *Food Sci Nutr* 2024, *12,* 345–360. doi:10.1002/fsn3.3980.

[16]Wang, R.; Chen, S.; Zheng, B.; Li, F.; Mao, L.; Liu, X.; Zhang, M. Polysaccharide–Protein Interactions and Their Application in Dysphagia Diet: A Review. *Food Rev Int* 2023, *39*, 1610–1632. doi:10.1080/87559129.2021.1963973.

[17] Lertstianboon, P.; Taniuchi, S.; Kajiwara, K.; Chaiseri, S. Effect of texture and viscosity on sensory attributes and palatability of IDDSI Level 4 and 5 foods for dysphagia patients. *J Texture Stud* 2022, *53*, 827–840. doi:10.1111/jtxs.12702.

3-2F

Click here to access/download;Figure;figure 3-2 F.tif

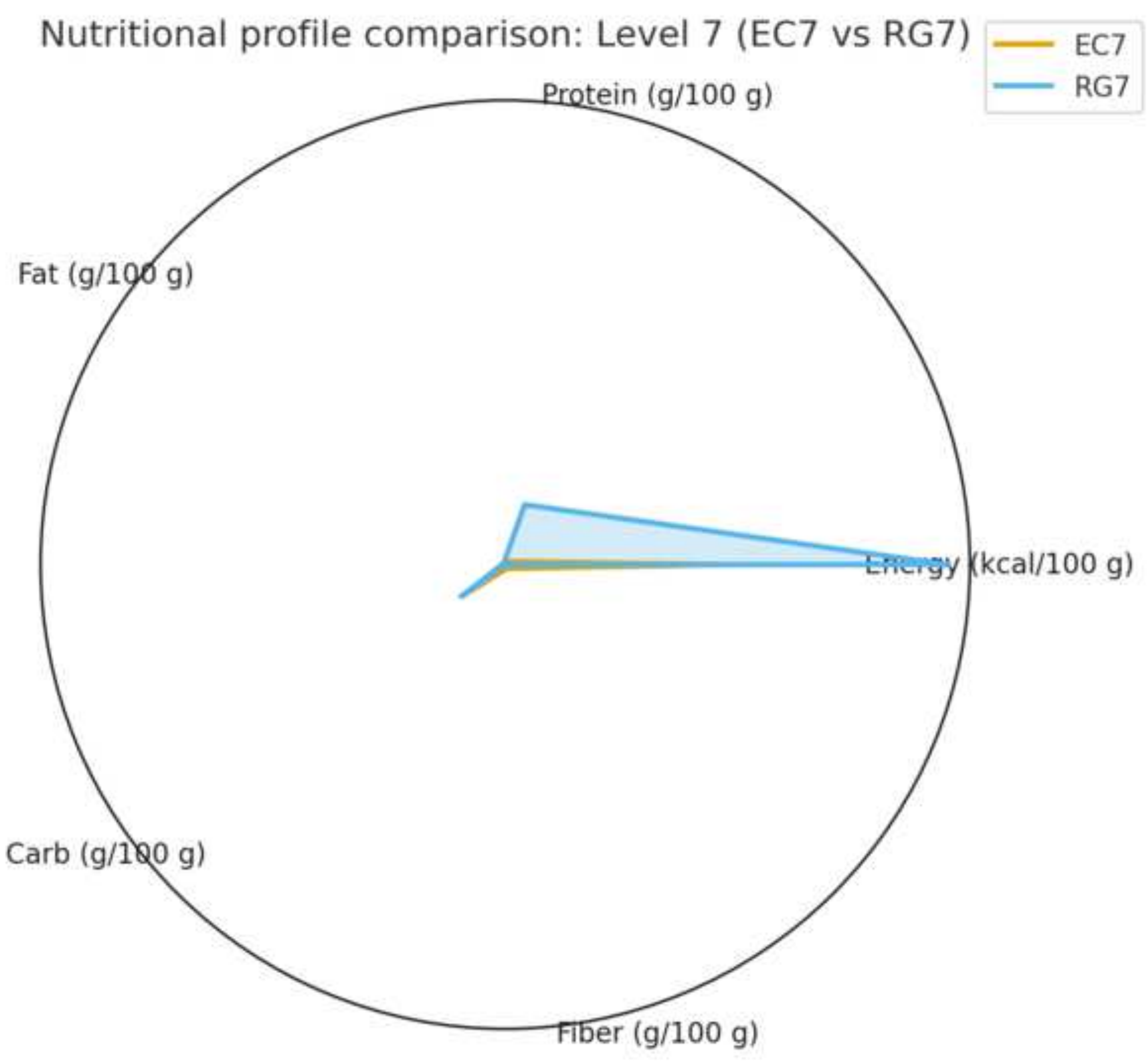

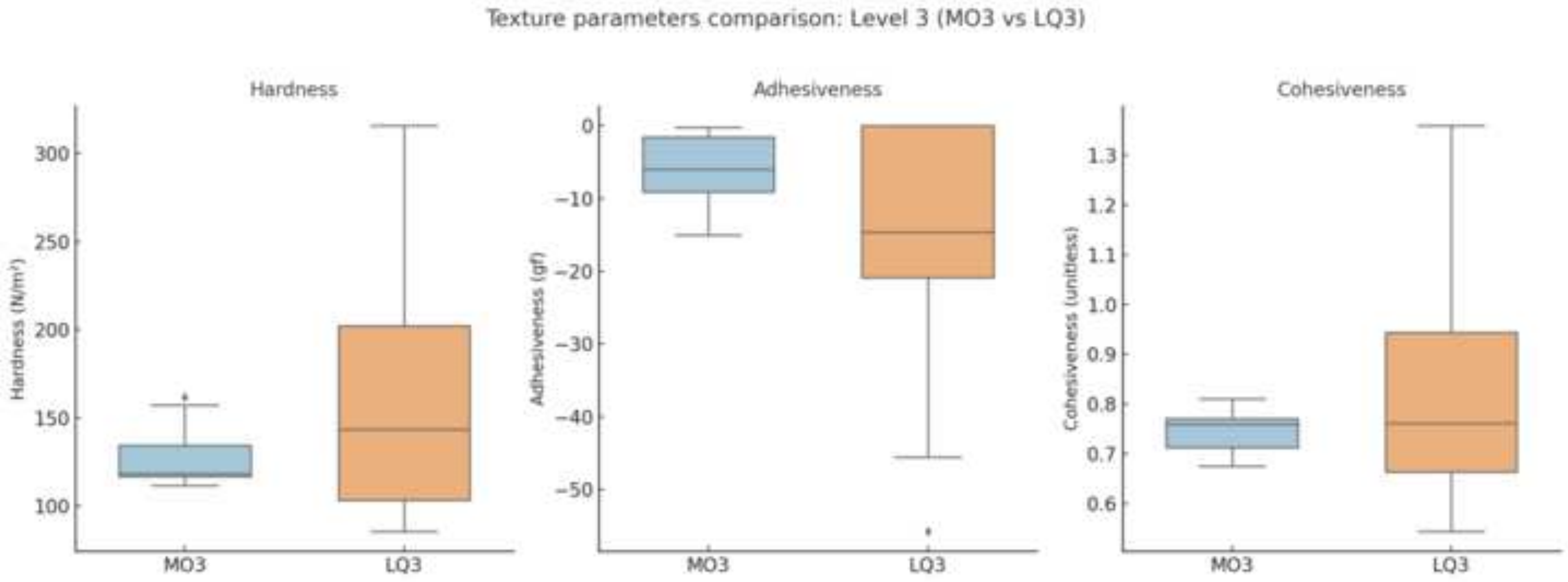
Texture parameters comparison: Level 3 (MO3 vs LQ3)
Hardness
Hardness (N/m²)
300
250
200
150
100
MO3
LQ3
Adhesiveness
Adhesiveness (gf)
0
−10
−20
−30
−40
−50
MO3
LQ3
Cohesiveness
Cohesiveness (unitless)
1.3
1.2
1.1
1.0
0.9
0.8
0.7
0.6
MO3
LQ3

3-2B

Click here to access/download;Figure;figure 3-2B.tif

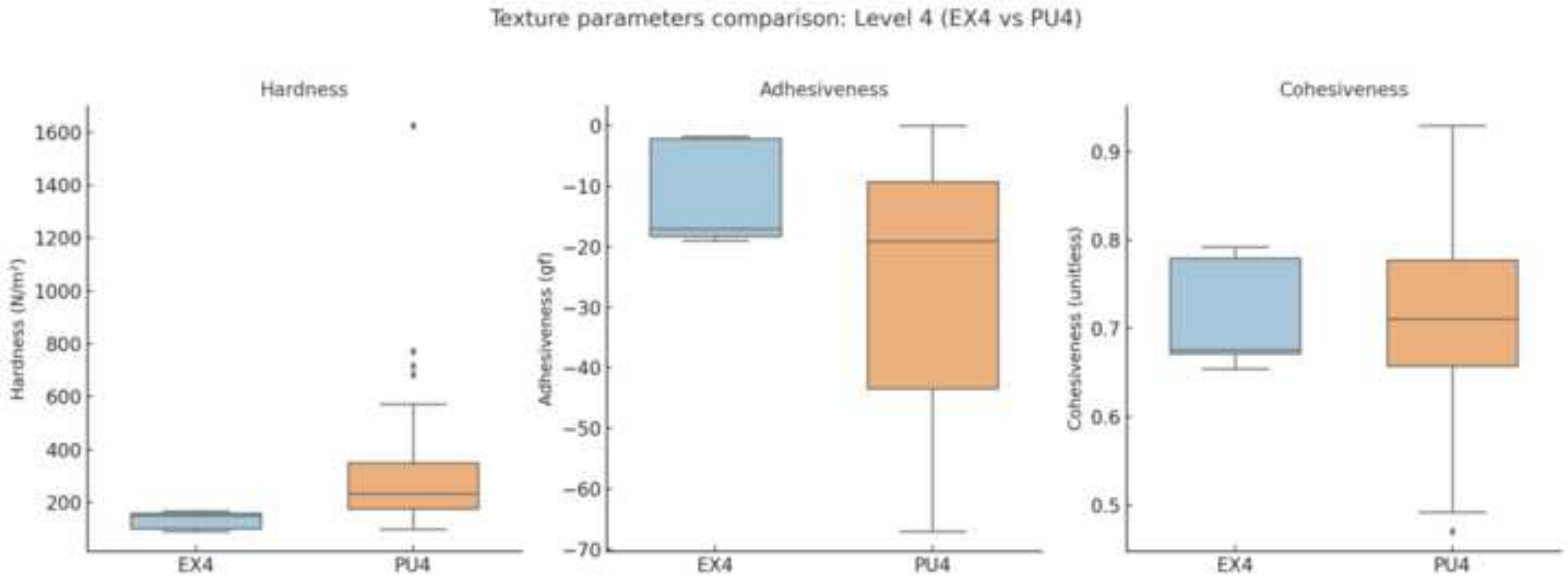

3-2C

Click here to access/download;Figure;figure 3-2C.tif

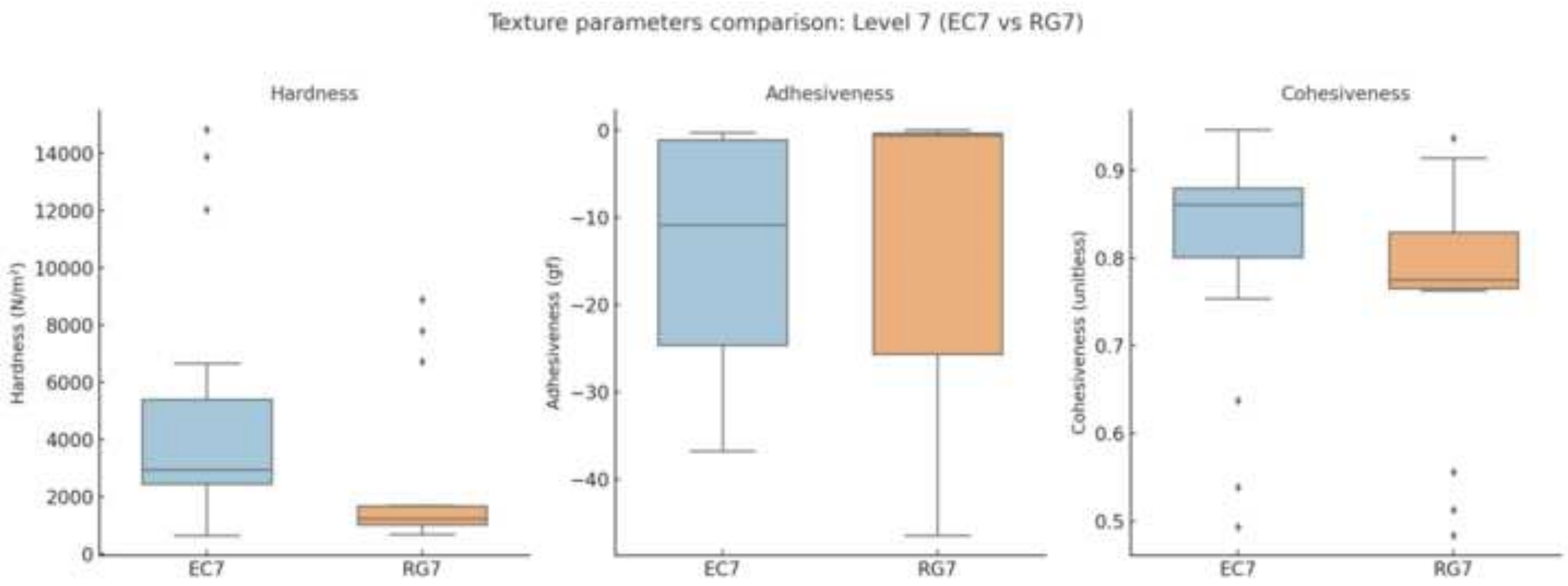

3-2E

Click here to access/download;Figure;figure 3-2E.tif

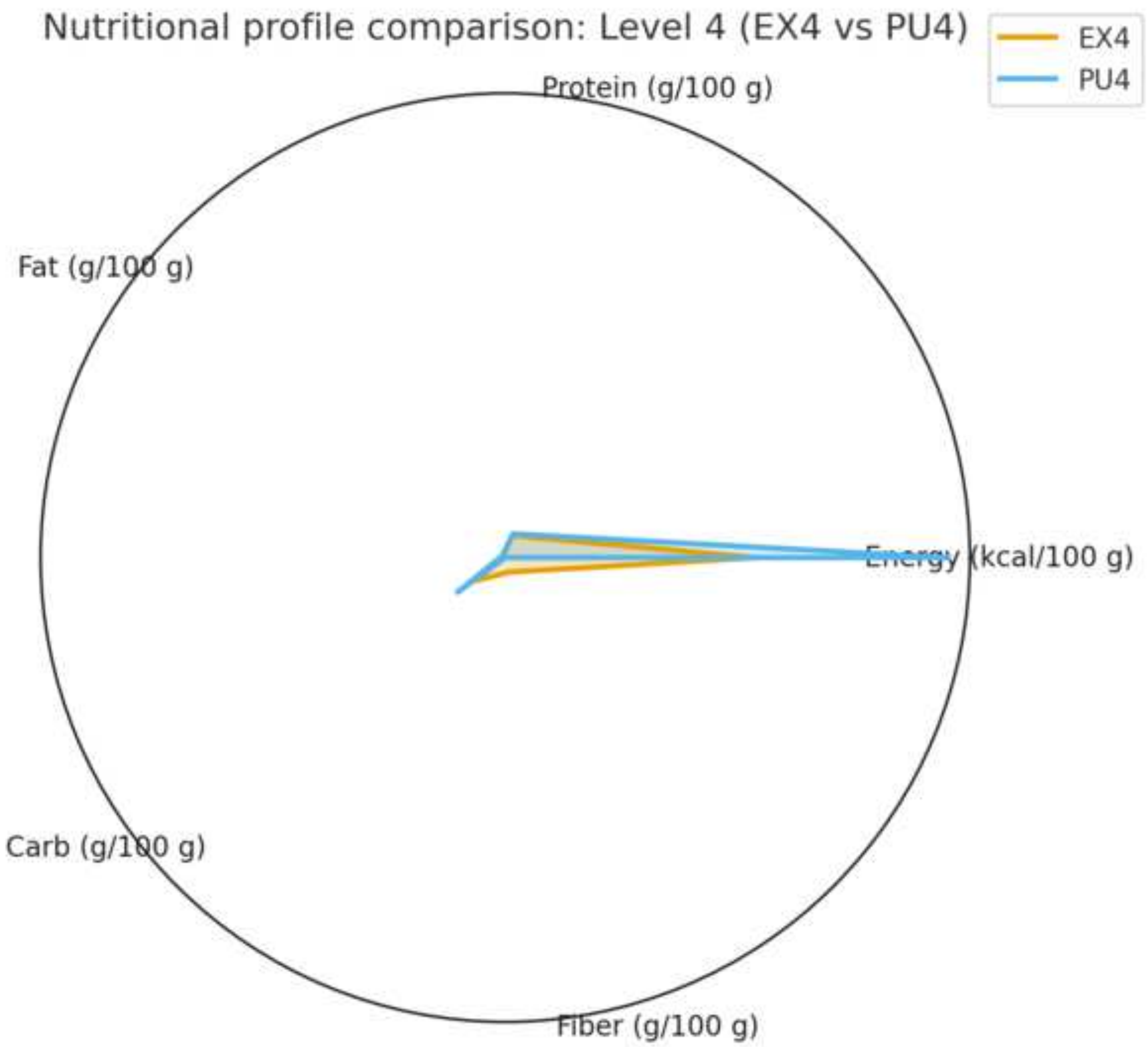

3-3D

Click here to access/download;Figure;figure 3-3 D.tif

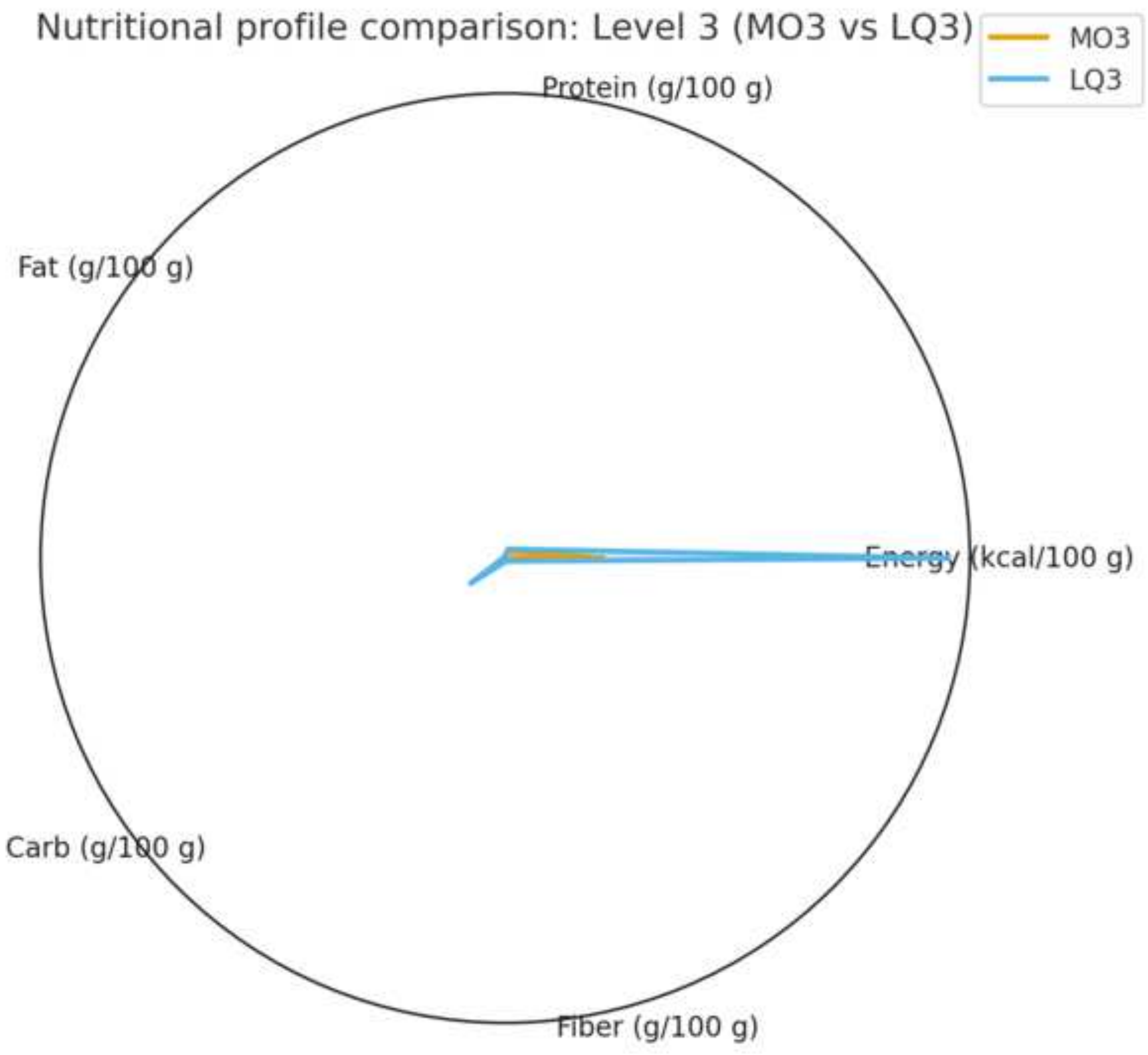

3-3A

Click here to access/download;Figure;figure 3-3A.tif

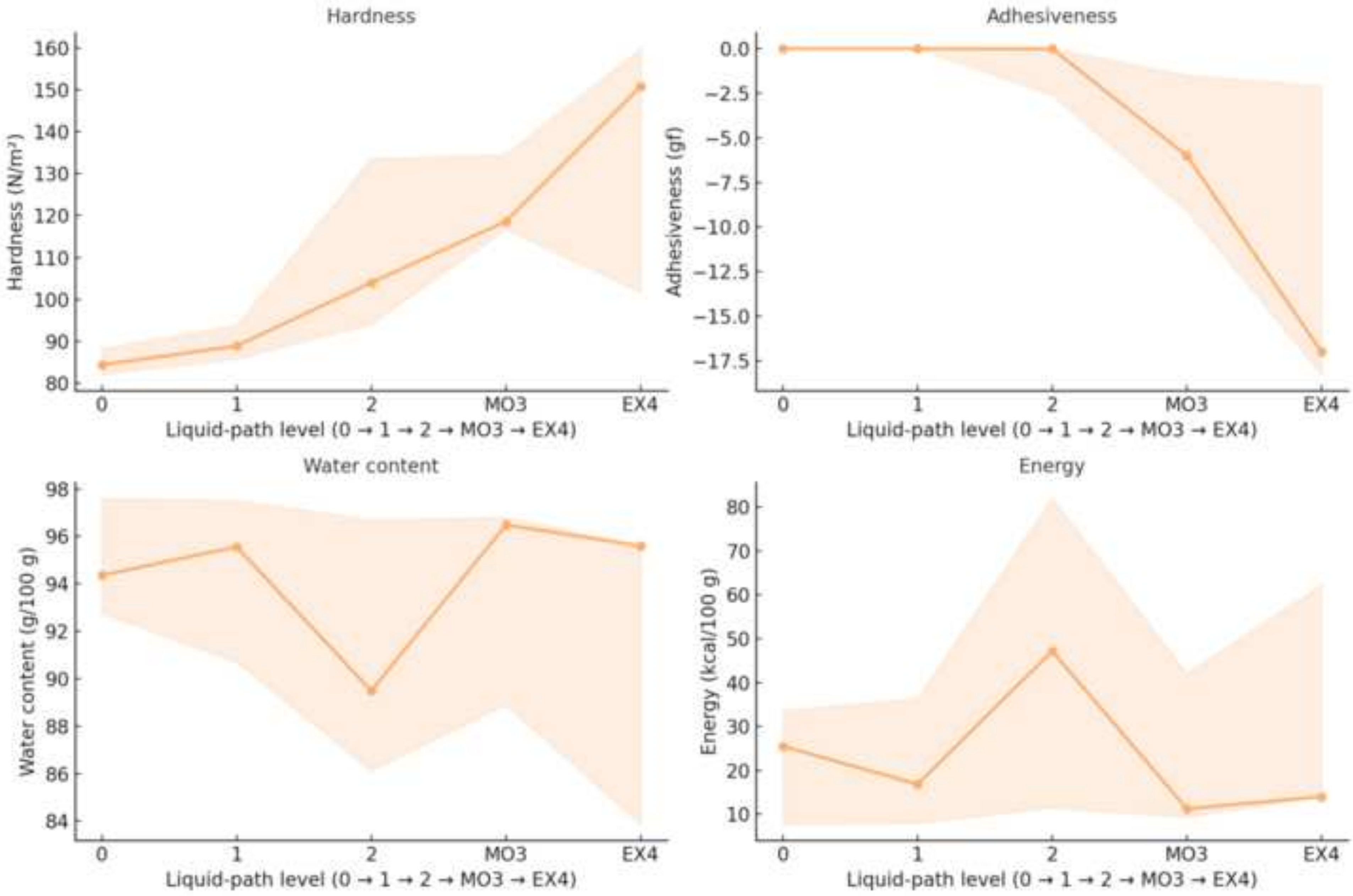

Click here to access/download;Figure;figure 3-3B.tif

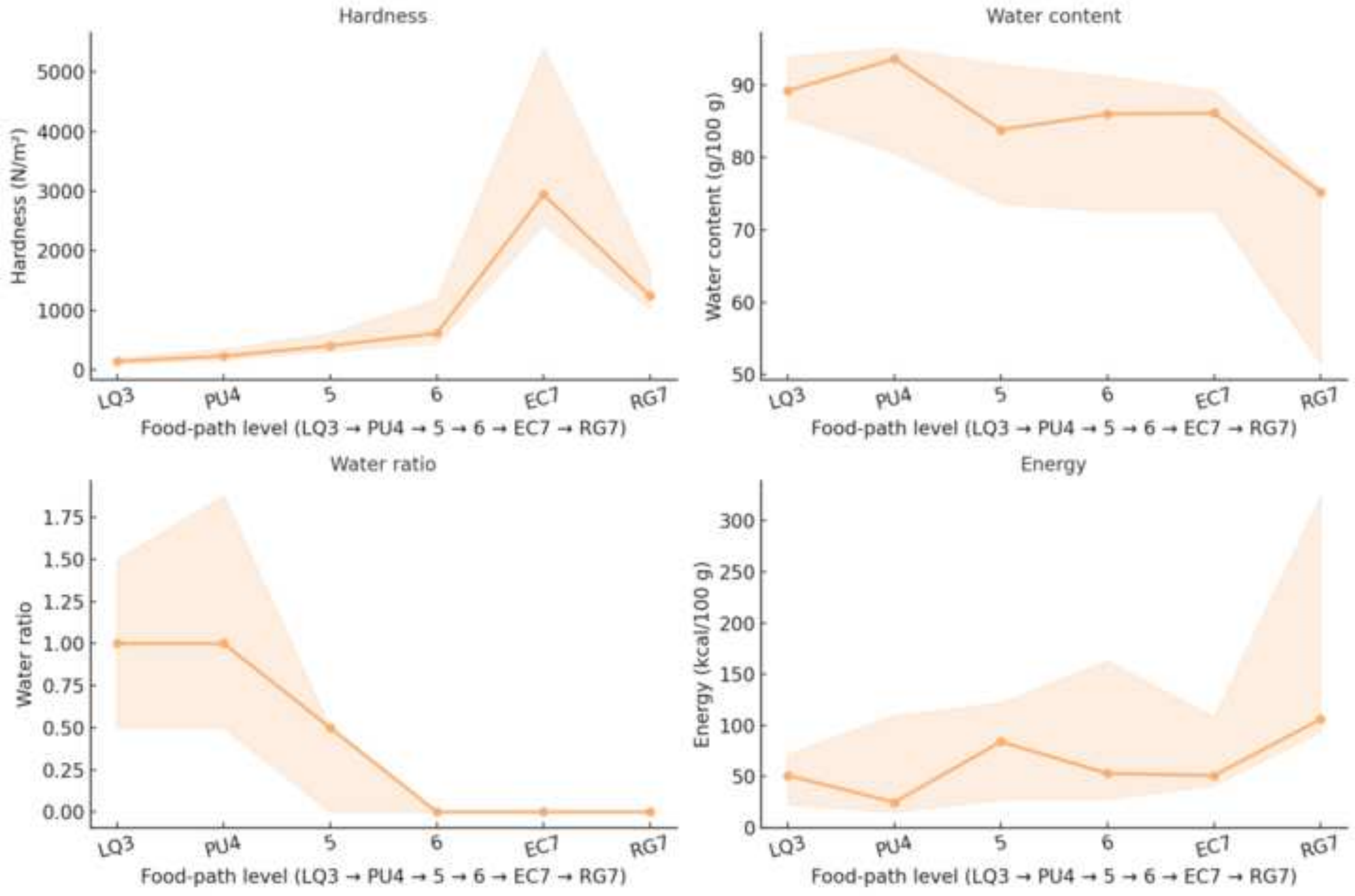

2-1

Click here to access/download;Figure;figure2-1.tif

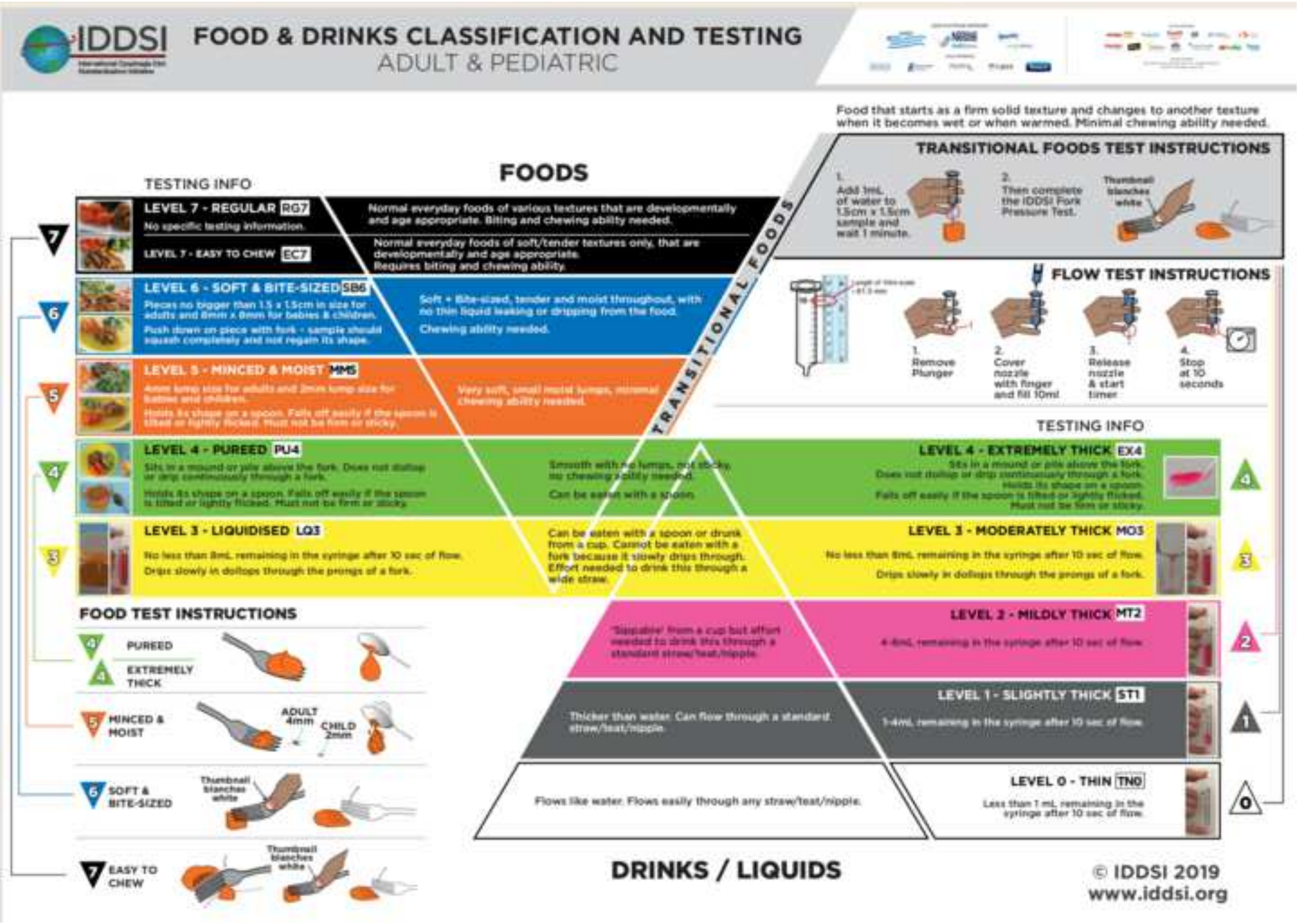

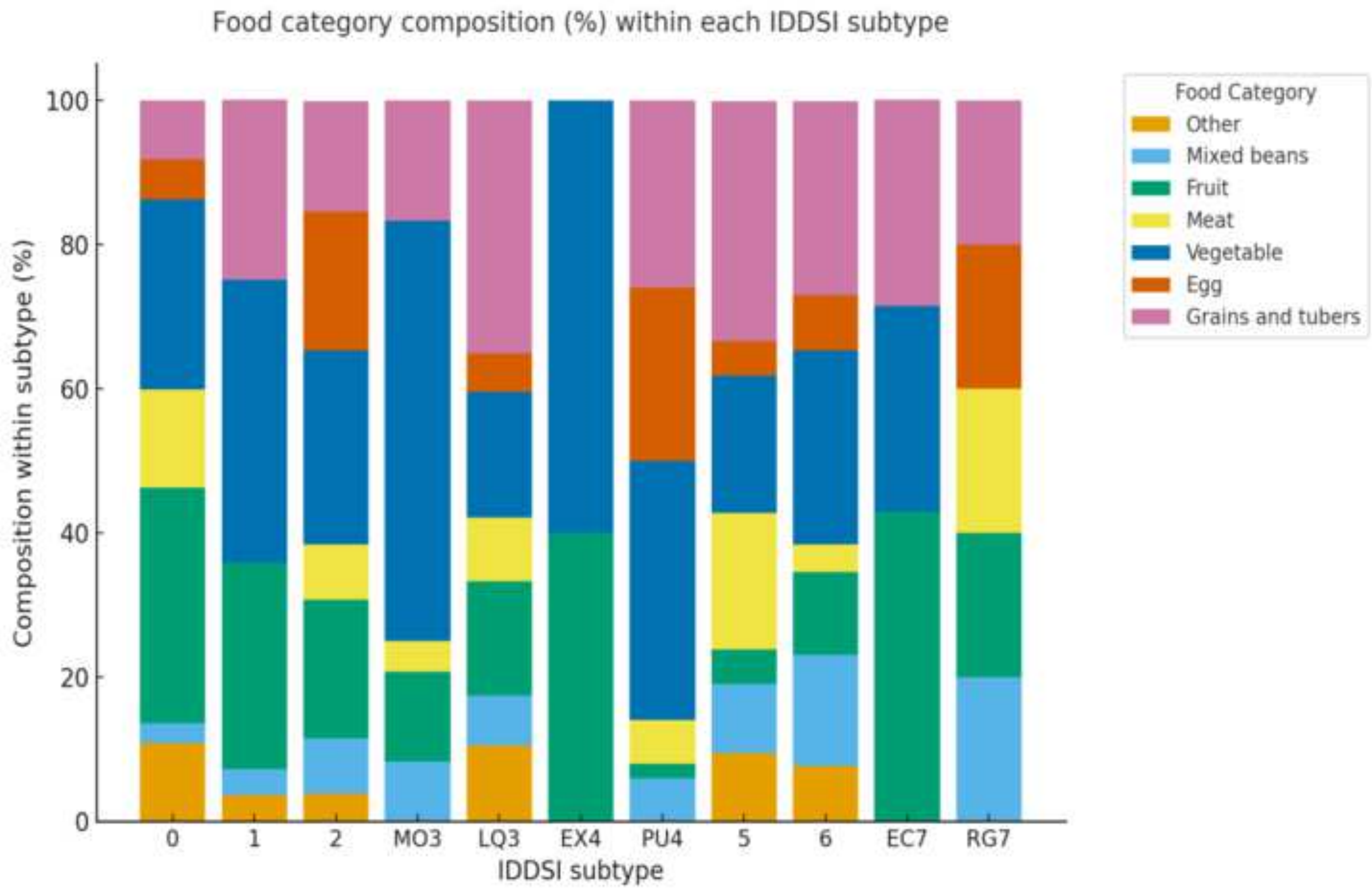

Food category composition (%) within each IDDSI subtype
Composition within subtype (%)
0
20
40
60
80
100
0
1
2
MO3
LQ3
EX4
PU4
5
6
EC7
RG7
IDDSI subtype
Food Category
Other
Mixed beans
Fruit
Meat
Vegetable
Egg
Grains and tubers
